# How Bacterial Chemoreceptors Evolve Novel Ligand Specificities

**DOI:** 10.1128/mBio.03066-19

**Published:** 2020-01-21

**Authors:** José Antonio Gavira, Vadim M. Gumerov, Miriam Rico-Jiménez, Marharyta Petukh, Amit A. Upadhyay, Alvaro Ortega, Miguel A. Matilla, Igor B. Zhulin, Tino Krell

**Affiliations:** a Laboratory of Crystallographic Studies, IACT (CSIC-UGR), Armilla, Spain; b Department of Microbiology, The Ohio State University, Columbus, Ohio, USA; c Department of Environmental Protection, Estación Experimental del Zaidín, Consejo Superior de Investigaciones Científicas, Granada, Spain; d Biology Department, Presbyterian College, Clinton, South Carolina, USA; e Emory Vaccine Center, Yerkes National Primate Research Center, Atlanta, Georgia, USA; Yale School of Medicine

**Keywords:** cache domains, chemotaxis, evolution, ligands, signal transduction

## Abstract

Chemoreceptor-based signaling pathways are among the major modes of bacterial signal transduction, and Pseudomonas aeruginosa PAO1 is an important model to study their function. Of the 26 chemoreceptors of this strain, PctA has a broad ligand range and responds to most of the proteinogenic amino acids, whereas PctB and PctC have a much narrower range and show strong ligand preference for l-glutamine and γ-aminobutyrate, respectively. Using several comparative genomics approaches, we show that these receptors are paralogs: *pctA* gene duplication in the common ancestor of the genus *Pseudomonas* led to *pctC*, whereas *pctB* originated through another, independent *pctA* duplication in the common ancestor of P. aeruginosa. Thus, the broad-range amino acid chemoreceptor was evolutionarily older, and chemoreceptors that complemented “missing” amino acid sensing abilities arose later in specific *Pseudomonas* lineages. Using comparative sequence analysis, newly solved crystal structures of PctA, PctB, and PctC ligand-binding domains, and their molecular dynamics simulations, we identified a conserved amino acid recognition motif and changes in the ligand-binding pocket that led to novel ligand specificities. In addition, we determined major forces driving the evolution of this group of chemoreceptors.

## Abstract (precis)

Many bacteria possess a large number of chemoreceptors that recognize a variety of different compounds. More than 60% of the genomes analyzed in this study contain paralogous chemoreceptors, suggesting that they emerge with high frequency. We provide first insight on how paralogous receptors have evolved and show that two chemoreceptors with a narrow ligand range have evolved from an ancestral protein with a broad chemoeffector spectrum. Protein structures show that multiple changes in the ligand-binding site account for the differences in the ligand spectrum. This work lays the ground for further studies aimed at establishing whether the principles of ligand-binding evolution reported here can be generalized for a wider spectrum of sensory proteins in bacteria.

## INTRODUCTION

Bacteria need to constantly adapt to changing environmental conditions to ensure survival. Among the different adaptational strategies is the ability of many bacteria to perform chemotaxis, a behavior based on the action of chemosensory pathways (1, 2). Typically, chemoeffector recognition at the ligand-binding domain (LBD) of chemoreceptors initiates the chemosensory signaling process (3). Bacteria have evolved chemotactic behavior for an enormous variety of compounds, such as different carbon, nitrogen, or energy sources; compounds that serve as final electron acceptors or inorganic ions; or compounds that inform the bacteria about their environment, such as plant hormones or quorum-sensing molecules (4, 5). To sense this variety of chemicals, many bacteria have evolved a large number of different chemoreceptors—in some cases more than 80 (6, 7). In order to recognize different chemoeffectors, chemoreceptors employ many different types of LBDs (5). Significant efforts are under way to determine chemoreceptor function, because ligand specificity for the vast majority of them remains unknown. Furthermore, there is little information available on how particular sets of chemoreceptors within a given species have evolved.

We have addressed this question here using Pseudomonas aeruginosa PAO1 as a model. This strain has 26 chemoreceptors, and approximately half of them have been functionally characterized (8, 9) (see Fig. S1 in the supplemental material). P. aeruginosa has 5 chemoreceptors with a dCache LBD, which is the most abundant LBD in bacteria (10) and is also found in eukaryotes (11). dCache domain-containing chemoreceptors recognize a variety of different compounds, such as proteinogenic amino acids (12), γ-aminobutyrate (GABA) (13), quaternary amines (14), purines (15), histamine and polyamines (8), taurine (16), and citrate (17). This family of chemoreceptors forms the primary family for amino acid chemotaxis (18), and its members show a wide phylogenetic distribution and have been identified in *Pseudomonas* (19–22), *Bacillus* (12, 17), *Vibrio* (23, 24), Helicobacter pylori (25), Campylobacter jejuni (26), Sinorhizobium meliloti (14), and the archaeon Halobacterium salinarum (27).

10.1128/mBio.03066-19.2 FIG S1 Schematic representation of Pseudomonas aeruginosa PAO1 chemoreceptors. The black segments are transmembrane regions, dark gray segments indicate HAMP (*h*istidine kinases, *a*denyl cyclases, *m*ethyl-accepting proteins, and *p*hosphatases) domains, and the light gray segments show the different types of the MCP (methyl-accepting chemotaxis protein) signaling domains. The effectors for characterized chemoreceptors as well as their binding mode are indicated. Download FIG S1, PDF file, 0.3 MB. Copyright © 2020 Gavira et al. 2020 Gavira et al. This content is distributed under the terms of the Creative Commons Attribution 4.0 International license.

P. aeruginosa PAO1 is strongly attracted to all 20 l-amino acids (28), which is enabled by the concerted action of the three dCache-containing chemoreceptors PctA, PctB, and PctC (21). The genes encoding these chemoreceptors are in close proximity (Fig. S2A), suggesting that they are a result of gene duplication. PctA binds and mediates chemoattraction to most of the proteinogenic amino acids (20, 21, 29). In contrast, PctB and PctC have a much narrower ligand spectrum (21) and recognize preferentially l-glutamine and GABA (29), respectively. The magnitude of the signaling input into these three receptors, as expressed by the ligand-receptor binding constant, was shown to determine the magnitude of output (13, 18).

10.1128/mBio.03066-19.3 FIG S2 The chemoreceptors PctA, PctB, and PctC of P. aeruginosa PAO1. (A) Organization and environment of the *pctA*, *pctB*, and *pctC* genes according to the *Pseudomonas* database (http://www.pseudomonas.com). (B) Sequence alignment. The transmembrane regions are shaded in yellow and flank the ligand-binding domains. Download FIG S2, PDF file, 0.4 MB. Copyright © 2020 Gavira et al. 2020 Gavira et al. This content is distributed under the terms of the Creative Commons Attribution 4.0 International license.

We report here the evolutionary history of Pct paralogs and report the three-dimensional (3D) structures of the LBDs from PctA, PctB, and PctC chemoreceptors in complex with different chemoattractants.

## RESULTS

### PctA is a predecessor of PctB and PctC.

**FIG 1:**
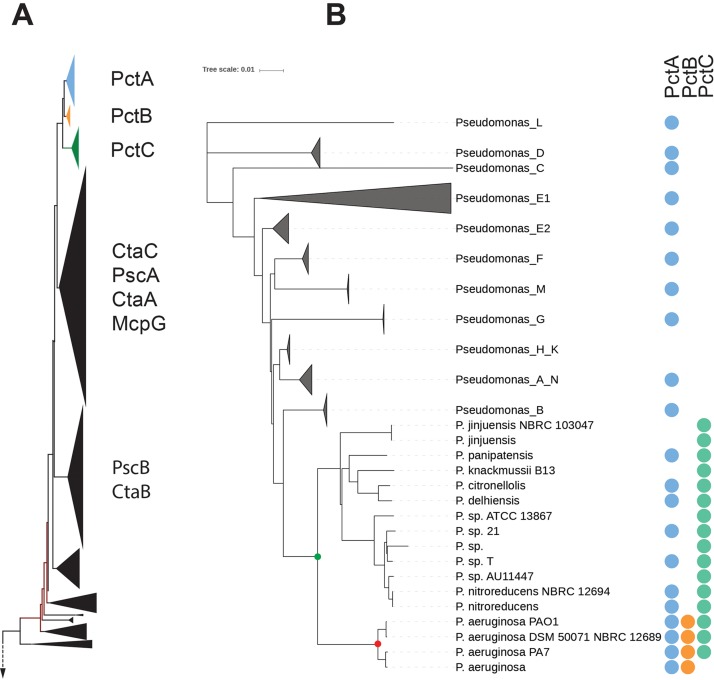
Evolutionary history of PctA homologs. (A) A fragment of the maximum-likelihood phylogenetic tree containing PctA, PctB, PctC, and their closest homologs. Positions of experimentally studied homologs are indicated. (B) Phyletic distribution of PctA, PctB, and PctC orthologs. The genome tree of *Pseudomonas* based on 120 conserved genes was generated from the Genome Taxonomy Database, and the presence of PctA, PctB, and PctC orthologs in corresponding genomes is shown by circles.

PctA, PctB, and PctC are assumed to be paralogs (21, 29), because they are encoded in the same genomic neighborhood and have a high sequence identity (21), which suggests recent gene duplication(s). However, paralogous relationships have not been clearly established; therefore, we inferred the evolutionary history of these proteins using a phylogenetic approach. We searched the bacterial nonredundant database with PctA, PctB, and PctC as queries using BLAST and collected 5,200 similar sequences that were clustered based on coverage and identity to remove redundancy (see Materials and Methods). The 2,143 sequences of the final set were aligned and used to construct a maximum likelihood phylogenetic tree. PctA, PctB, and PctC orthologous clusters formed a distinct group at the top of the tree (Fig. 1A). Orthologous relationships were further verified using the bidirectional best-BLAST-hit approach. Comparative analysis showed that many bacterial genera that lack PctB and PctC contain PctA orthologs (see Data Set S1A in the supplemental material). Further comparative analysis and phylogenetic profiling based on the latest bacterial taxonomy (30) revealed that PctA is a predecessor of both PctB and PctC and is present in all representatives of the family *Pseudomonadaceae*, while PctC is present only in the genus *Pseudomonas* (Fig. 1B). Remarkably, PctB orthologs were identified in genomes of only one species—P. aeruginosa. Taken together, our results indicate that *pctA* gene duplication in the common ancestor of the genus *Pseudomonas* led to the birth of *pctC*, whereas the *pctB* gene originated later through another, independent *pctA* duplication in the common ancestor of P. aeruginosa.

10.1128/mBio.03066-19.10 DATA SET S1 Bioinformatics data. (A) Presence of PctA homologs in different *Pseudomonadaceae* members. (B) Genetic alterations of the *pctA*, *pctB*, and *pctC* genes and in their neighborhoods in P. aeruginosa strains. (C) Presence of *pctA*, *pctB*, and *pctC* genes in different P. aeruginosa complete and draft genomes. (D) Abundance of PctA homologs in different bacterial strains. Download Data Set S1, XLSX file, 0.1 MB. Copyright © 2020 Gavira et al. 2020 Gavira et al. This content is distributed under the terms of the Creative Commons Attribution 4.0 International license.

### Sensory domains of PctA homologs are rapidly evolving.

Phylogenetic profiling revealed that PctA and its homologs are subject not only to gene duplication but also to gene loss (Fig. 1B). To further understand the scale and frequency of these major evolutionary events, we performed comparative genome analysis across more than 200 P. aeruginosa genomes. This analysis revealed many changes in the neighborhoods of these genes, ranging from changing positions within the neighborhood to duplications, conversions to pseudogenes, and gene loss (Data Set S1B and C). Gene duplication appears to be the major driving force in the evolution of PctA. We detected numerous events of *pctA* duplication in the family *Pseudomonadaceae* and among their closest relatives (Data Set S1D). For example, genomes of P. fluorescens NCIMB 11764 and Pseudomonas pseudoalcaligenes S1 contain nine and eight PctA homologs, respectively (Data Set S1D). Distribution of PctA homologs across different clusters on the PctA homolog tree (Fig. S3) reveals that these are independent events that occurred prior to more recent duplications resulting in the birth of PctC and PctB.

10.1128/mBio.03066-19.4 FIG S3 Clusters of PctA homolog sequences paired with corresponding sequence logos of ligand-binding pocket regions. Regions of sequence logos with alterations in the binding motif are highlighted in turquoise. I, P. aeruginosa PAO1; II, Pseudomonas fluorescens Pf0-1; III, Pseudomonas syringae pv. tomato strain DC3000; IV, Pseudomonas putida KT2440; V, Vibrio cholerae O1 bv. El Tor strain N16961. The closest homolog of McpU in our data set is ABZ00079.1 (86% identity) in Pseudomonas putida GB-1; the closest homolog of TlpQ is KFX69128.1 (66%) in Pseudomonas taeanensis MS-3. Download FIG S3, PDF file, 0.2 MB. Copyright © 2020 Gavira et al. 2020 Gavira et al. This content is distributed under the terms of the Creative Commons Attribution 4.0 International license.

Full-length sequence comparison showed that PctA, PctB, and PctC proteins have an average sequence identity above 77%. However, a striking difference was observed between their sensory and signaling domains. The average sequence identity was more than 96% in the signaling domain but less than 56% in the dCache_1 sensory domain (Fig. S2B). Similar results were obtained by comparing PctA orthologs from representatives of all genera in the family *Pseudomonadaceae* (Data Set S1D).

Taken together, our results demonstrate that chemoreceptors of the PctA type are in the active stage of evolutionary changes driven by gene duplication and accelerated evolution of their sensory domains. In order to understand structure-function relationships within these rapidly evolving sensors, we solved and analyzed their structures with corresponding ligands and examined patterns of conservation in their ligand-binding pockets. The results enabled us to identify a conserved motif, which is critical for amino acid binding, and regions that determine a specific amino acid repertoire of each sensor.

### The 3D structures of the LBDs of PctA, PctB, and PctC in complex with their respective ligands.

We report here crystal structures of PctA/B/C-LBD in complex with 6 different ligands. The three PctA-LBD structures contain bound l-Met, l-Trp, and l-Ile; PctB-LBD was determined in complex with l-Gln and l-Arg, whereas PctC-LBD has bound GABA.

**FIG 2:**
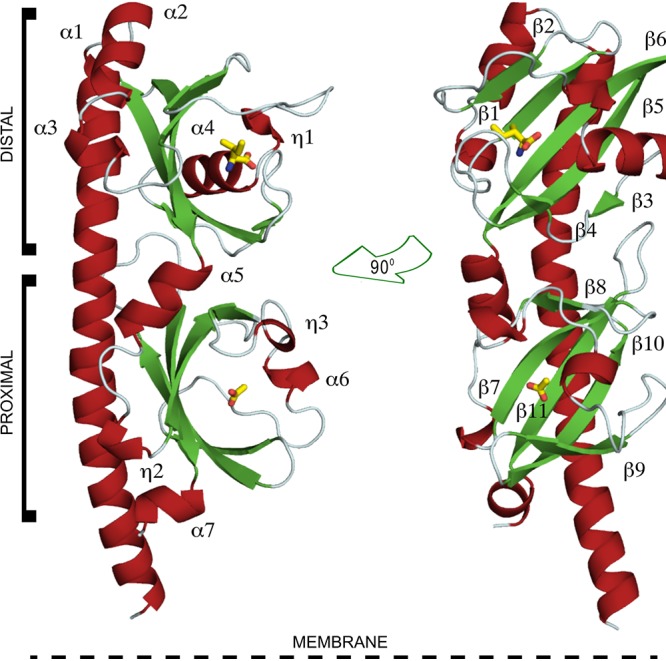
Ribbon diagram of the PctA-LBD structure. Secondary structural elements are labeled. l-Ile and acetate, bound at the distal and proximal modules, respectively, are shown in stick mode.

The overall structure of the three paralogs is illustrated in Fig. 2 on the PctA-LBD/l-Ile structure that is composed of a long N-terminal helix followed by two stacked α/β modules. The asymmetric units of crystals of PctA-LBD and PctB-LBD contain two chains that contain bound amino acid in the membrane-distal module (Fig. S4). In addition, PctA-LBD contained acetate in the membrane-proximal module. Microcalorimetric titrations of PctA-LBD with 20 mM acetate did not show binding, indicating that acetate, present in the crystallization buffer at 100 mM, binds with very low affinity.

10.1128/mBio.03066-19.5 FIG S4 The overall structure of PctA-LBD, PctB-LBD, and PctC-LBD. (A) Ribbon diagrams of the crystallographic dimers present in the asymmetric units of PctA-LBD and PctC-LBD. Chains A and C are shown for PctC-LBD. Bound ligands are shown in purple. (B) Topological organization of secondary structural elements of the three paralogous structures. Download FIG S4, PDF file, 0.2 MB. Copyright © 2020 Gavira et al. 2020 Gavira et al. This content is distributed under the terms of the Creative Commons Attribution 4.0 International license.

**FIG 3:**
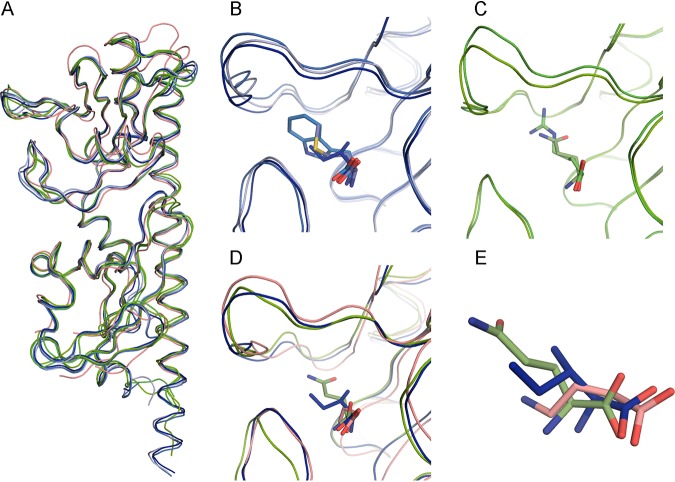
Structural alignment of the ligand-binding domains of PctA, PctB, and PctC. (A) Alignment of all six structures. PctA-LBD, shades of blue; PctB-LBD, shades of green; PctC-LBD, pink. (B and C) Expanded view of the binding pockets of PctA-LBD and PctB-LBD, respectively, containing different ligands. (D) Expanded view of the superimposed binding pockets of the three paralogous receptors. (E) Position of l-Ile, l-Gln, and GABA in the superposition of the three LBDs. The ligand carbon color corresponds to that of the corresponding protein chain.

PctC-LBD crystallized in a different space group with 7 chains in the asymmetric unit, of which only 2 contained GABA inside the binding pocket of the membrane-distal module. The overall structures of the three paralogous proteins are similar (Fig. S4), and their superimposition showed a close alignment (Fig. 3A), with PctA-LBD being more similar to PctB-LBD and less to PctC-LBD (Table S1A). The secondary structures of the membrane-distal modules were highly similar, whereas significant changes were detected in the membrane-proximal modules (Fig. S4B).

10.1128/mBio.03066-19.7 TABLE S1 Structural alignment of PctA/B/C-LBD structures. (A) Alignment of structures among each other. (B) Alignment with homologous structures deposited in the PDB data bank. (C) Relative positions of the four amino acids conserved in the ligand-binding pocket of the three paralogs. Download Table S1, PDF file, 0.2 MB. Copyright © 2020 Gavira et al. 2020 Gavira et al. This content is distributed under the terms of the Creative Commons Attribution 4.0 International license.

We have conducted molecular dynamics (MD) simulations to measure the extension of the different binding pockets. Data show that the binding sites are within the margin of error of very similar size (Text S1). On the other hand, significant differences in the orientations of ligands within the binding pockets of PctA, PctB, and PctC were detected (Text S1). A structural alignment of PctA-LBD with all PDB entries showed that it is particularly similar to chemoreceptors Mlp37 and Mlp24 of Vibrio cholerae, which also bind amino acids (Table S1B).

10.1128/mBio.03066-19.1 TEXT S1 Study of the size of the ligand-binding pocket and ligand orientation in PctA/PctB/PctC-LBD structures based on MD simulations. Download Text S1, PDF file, 0.4 MB. Copyright © 2020 Gavira et al. 2020 Gavira et al. This content is distributed under the terms of the Creative Commons Attribution 4.0 International license.

### Evidence for ligand-induced structural changes.

**FIG 4:**
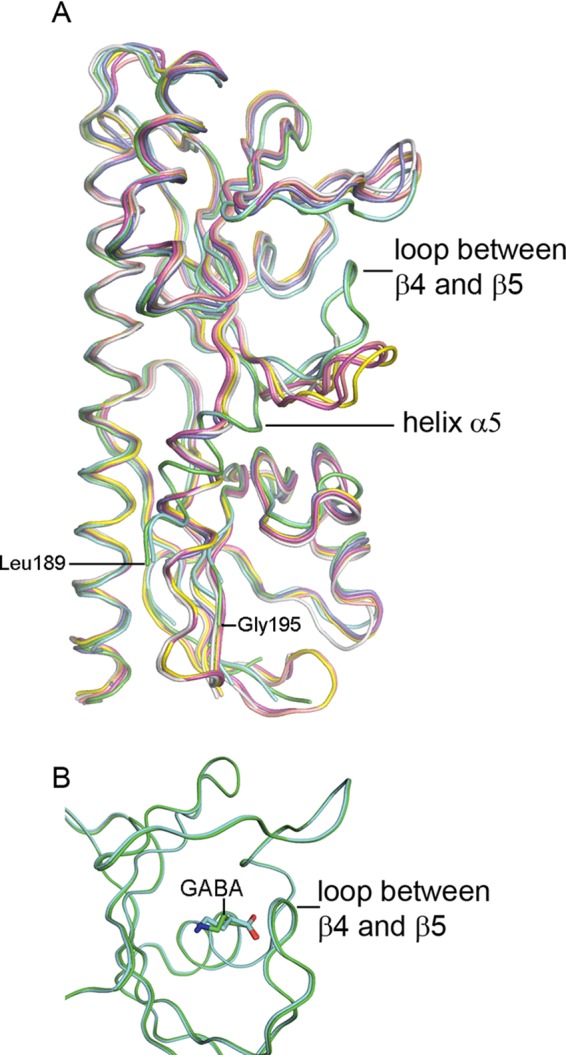
Evidence for ligand-induced structural changes in PctC-LBD. (A) Structural alignment of the seven PctC-LBD chains of the asymmetric unit. Green and cyan, GABA inside the binding pocket; pink, yellow, and salmon, GABA at the entrance of the binding pocket; gray, acetate in the binding pocket; blue, empty pocket. (B) Superimposition of the membrane-distal modules of the two PctC-LBD chains with GABA inside the binding pocket.

Ligand-induced structural changes have been observed for four-helix bundle domains (31, 32), but the knowledge of dCache domains is more sparse. Of the 7 chains in the PctC-LBD asymmetric unit, two contained GABA inside the binding pocket, whereas the binding pocket is empty or occupied by an acetate molecule in the remaining chains. The superimposition of these 7 chains (Fig. 4A) shows that the loop connecting β4 and β5 in the two GABA-containing chains has undergone an almost 90° movement and closes in over bound GABA (Fig. 4B). The position of this loop in the remaining 5 chains is very similar, indicating that the presence of GABA in the binding pocket has triggered this movement. This loop movement appears to displace helix α5, which connects the distal to the proximal module (Fig. 4A). In addition, no density was observed for residues Leu189 to Gly195 in the GABA-containing structures, indicating that this region is present in multiple conformations and that its disorganization may be related to stimulus transmission to the transmembrane part of the receptor. Differences in ligand occupation of the 7 protomers appear to be due to crystal packing. Whereas in the two chains that contain bound GABA, the loop connecting β4 and β5 either is solvent exposed or shows minor interactions with other chains, the corresponding loops in the remaining chains maintain extensive interactions with neighboring protomers.

### The molecular detail of ligand recognition.

**FIG 5:**
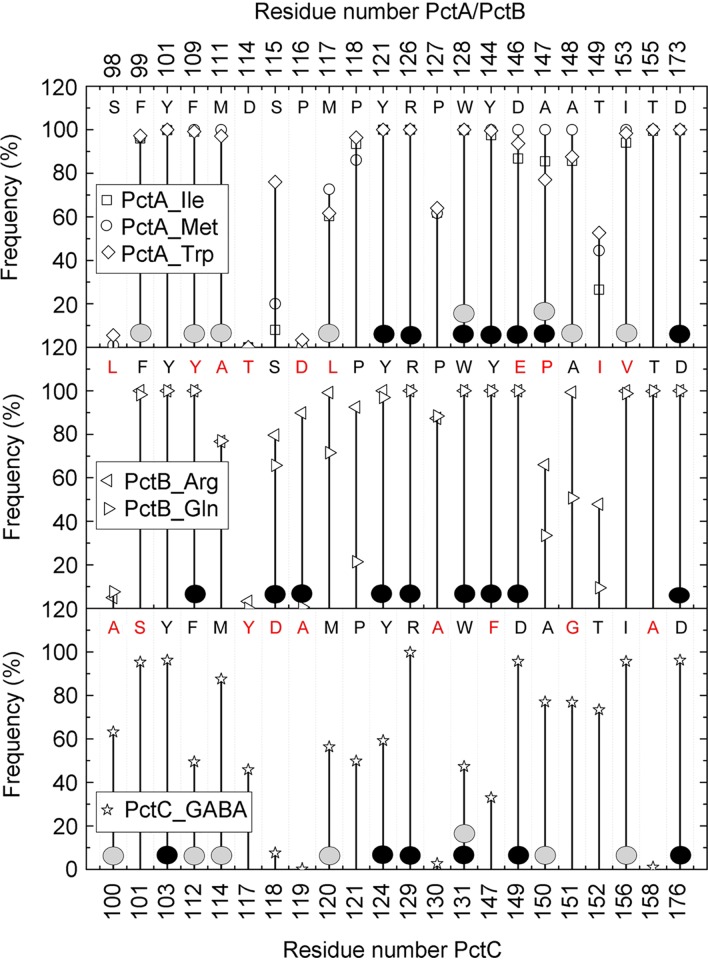
Amino acids involved in ligand binding. Probability of PctA, PctB, and PctC residues being within 5 Å of the bound ligand as derived from molecular dynamics studies. Residues involved in hydrogen bonding and hydrophobic interactions with ligands are marked as solid circles in black and gray, respectively. The amino acids for each protein are indicated in the upper part of each panel. The corresponding amino acids in PctB and PctC that are different from PctA are indicated in red.

**FIG 6:**
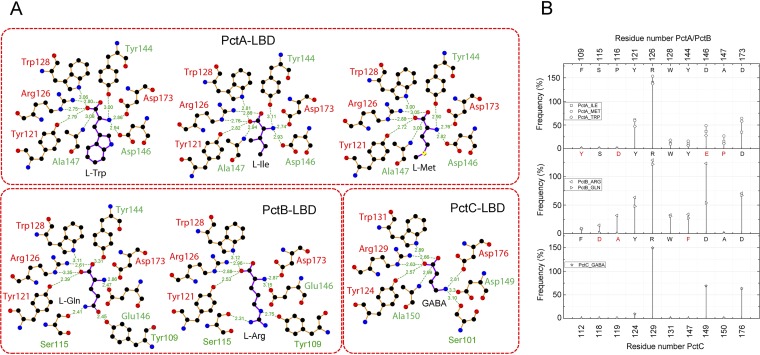
The hydrogen bonding network established between the three paralogous receptors and bound ligands. (A) The hydrogen bonding network as established by the inspection of the 3D structures. The figure was prepared using the LigPlot software (80). (B) Residues involved in hydrogen bond formation as derived from molecular dynamics simulations. The amino acids for each protein are indicated in the upper part of each panel. The corresponding amino acids in PctB and PctC that are different from PctA are indicated in red.

The inspection of electron densities in the different binding pockets has permitted the placement of the different amino acids (Fig. S5), and their superimposition in the PctA-LBD and PctB-LBD structures shows that the conserved parts of bound amino acids are in a similar position (Fig. 3B to E). The GABA amino group is in a similar position as the α-amino group of ligands bound to PctA-LBD and PctB-LBD. To identify the residues that interact with the ligands, we performed short all-atom 10-ns MD simulations of the six ligand-bound structures, and Fig. 5 summarizes the probability of amino acid residues to be within 5 Å from the bound ligand. The hydrogen bonding network as derived from the analysis of the X-ray structures is shown in Fig. 6A, whereas Fig. 6B provides information on hydrogen bonding dynamics and shows the frequency at which hydrogen bonds are formed in MD simulations.

10.1128/mBio.03066-19.6 FIG S5 Amino acids present in the ligand-binding sites of the three paralogous receptors. Mesh representation of the final |2Fo–Fc| electron density map contoured at 1.0-σ level. Amino acid structures are shown in stick mode. Download FIG S5, PDF file, 0.3 MB. Copyright © 2020 Gavira et al. 2020 Gavira et al. This content is distributed under the terms of the Creative Commons Attribution 4.0 International license.

The inspection of all structures showed that amino acids from 22 different homologous positions interact with the bound ligand, and in the case of PctA, 19 amino acids interact with bound amino acids. The side chains of the bound amino acids form hydrophobic interactions with Phe99 and Tyr101 (β1) and with different residues on the segment 109 to 118, which corresponds to the C-terminal part of β2 and the following loop (Fig. 2; Fig. S4). The inspection of the structures shows that the carboxyl and amino groups of the amino acids are coordinated by an extensive hydrogen bonding network formed by 8 bonds (Fig. 6A), involving 7 different amino acids. MD simulations (Fig. 6B) reveal that Arg126 plays a central role and forms hydrogen bonds with high frequency (note: the frequency of approximately 150% indicates that this residue establishes two hydrogen bonds).

Only 12 of the 19 residues that interact with bound ligands in PctA-LBD are conserved in PctB-LBD. PctB has a strong ligand preference for l-Gln (*K_D_* [equilibrium constant] = 1.2 μM) but also binds l-Arg with an affinity of 64 μM (29). Both ligands contain side chains that may form hydrogen bonds, a fact that is also reflected in the molecular detail of their recognition. MD simulations indicate hydrogen bond formation between the l-Gln and/or l-Arg side chains with Tyr109, Ser115, and Asp116 (Fig. 6B). Structure inspection also shows that Tyr109 and Ser115 form hydrogen bonds, whereas Asp116 points away from bound ligand but, since it is located on the long loop connecting β2 and η1 (Fig. 2), may swing in to interact with the bound ligand. Structure inspection indicated that the hydrogen bonding network of PctB-LBD to coordinate the conserved part of bound amino acids is very similar to that of PctA-LBD (Fig. 6A). However, MD simulations show that the frequency of hydrogen bond formation is higher than that of PctA-LBD, indicative of a particular relevance of hydrogen bond formation in PctB.

As stated above, PctC has originated from an ancestral PctA in another evolutionary event, and 13 of the 19 amino acids involved in ligand binding in PctA are conserved in PctC. Figure 5 illustrates that the frequency of ligand-protein interaction in PctC is in general lower than that seen in PctA and PctB. As in PctA and PctB, Arg129(126) plays a central role in coordinating the ligand carboxylate group (Fig. 5 and 6). Another common feature of all three paralogs is that two residues with acidic side chains, Asp149 and Asp176 in PctC, interact with the ligand amino group. Hydrogen bonds seen in the X-ray structure involving PctC Ser101, Tyr124, Trp131, and Ala150 (Fig. 6A) are formed with a very low frequency in MD simulations (Fig. 6B). In none of the structures do bound ligands establish hydrogen bonds through bound water molecules. Four amino acids are conserved in the ligand-binding pockets of the three paralogs (colored red in Fig. 6A). In pairwise alignments of protein structures, these amino acids are in very similar positions, particularly in the alignment of PctA-LBD with PctB-LBD (Table S1C).

### Identification of a sequence pattern for amino acid binding.

**FIG 7:**
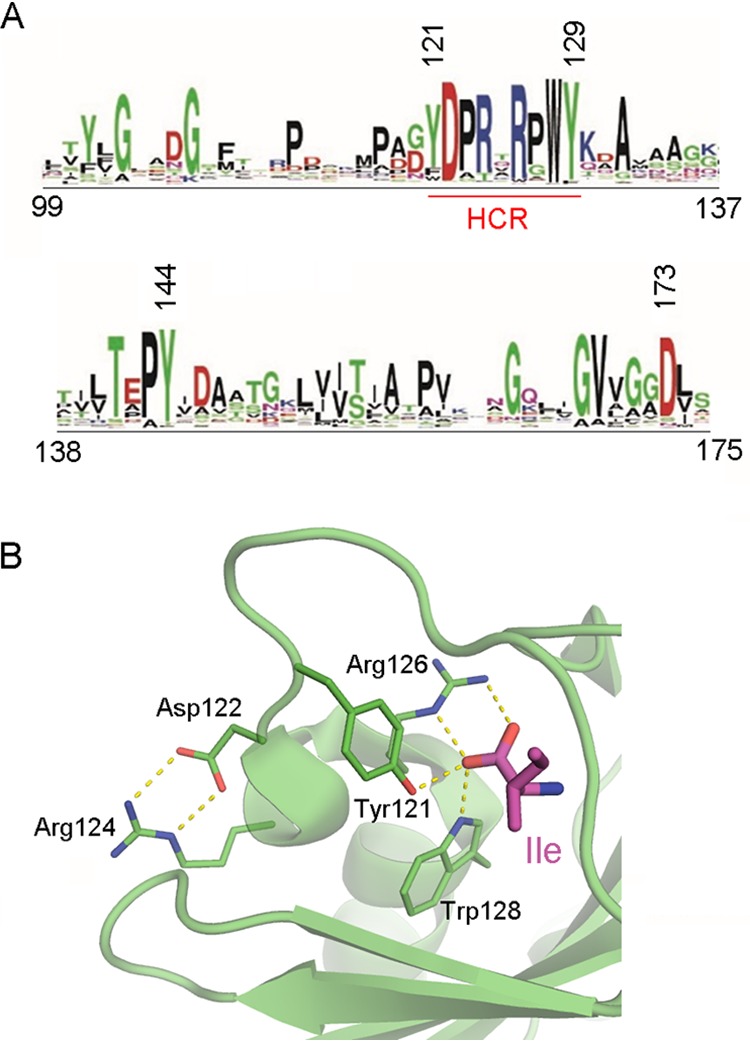
The conserved sequence motif for amino acid recognition. (A) The sequence logo was produced using all sequences except those of cluster L4 (see Fig. S3). Amino acid numbering is according to the PctA sequence. The highly conserved region (HCR) is underlined in red. (B) Expanded view of the binding pocket of PctA-LBD containing bound l-Ile. Conserved amino acids are shown in the stick mode.

Once the structures revealed the amino acids involved in ligand binding, we built sequence logos based on their multiple sequence alignments that were mapped on the tree (Fig. 1). Remarkably, we identified a highly conserved region (HCR) that was present in all the clusters, except L4. As indicated on the right side of Fig. S3, members of many clusters have been shown to bind different amino acids. In pseudomonads, so far three dCache_1 LBD-containing chemoreceptors have been identified that bind ligands other than amino acids, namely, the histamine and polyamine binding chemoreceptors McpU (19, 33) and TlpQ (8) as well as the purine chemoreceptor McpH (15). Interestingly, these chemoreceptors were all present in cluster L4 (Fig. S3), indicating that the HCR is specific for amino acids. We removed the sequences corresponding to L4 and built a sequence logo that is shown in Fig. 7A. The structural reasons for the conservation of this region are (i) the establishment of hydrogen bonds between three residues of this motif (Tyr121, Arg126, and Trp128) and the carboxyl moiety of the bound amino acid and (ii) the formation of a salt bridge between Asp122 and Arg124 that appears to be required for the formation of helix α1 (Fig. 7B).

### No evidence for ligand binding at the membrane-proximal modules.

In all three structures, amino acids are bound to the membrane-distal module, which raises the question of the function of the membrane-proximal module. To identify potential candidate molecules that may bind to this module, we have carried out *in silico* docking experiments with the 6 structures reported. Initial control experiments involved the docking of the 6 ligands to their cognate LBDs. The resulting extra precision (XP) scores (34) (Table S2) range from −13,74 to −9.37 kcal/mol and are highly significant.

10.1128/mBio.03066-19.8 TABLE S2 Glide XP docking scores obtained for PctA-LBD, PctB-LBD, and PctC-LBD for the best ligand candidates. Download Table S2, PDF file, 0.3 MB. Copyright © 2020 Gavira et al. 2020 Gavira et al. This content is distributed under the terms of the Creative Commons Attribution 4.0 International license.

Subsequently, the collection of all natural compounds (5,391) from the ZINC database was used for docking experiments with the membrane-proximal module. For all compounds, the absolute value of the docking scores was much lower than those of the ligands that bind to the membrane-distal module (Table S2). Top docking scores were −6.53 (PctA), −4.47 (PctB), and −6.05 (PctC). Since a docking score of −6 is considered the lower threshold for significance, the binding of some top-scoring compounds (Table S2) was verified by microcalorimetric titrations using purified PctA-LBD, in the presence and absence of bound amino acid. In all cases, an absence of binding was noted, suggesting that the membrane-proximal module may not be involved in small-molecule ligand binding.

## DISCUSSION

The number of chemoreceptors in different bacteria varies depending on the environment they inhabit (6). Even within the same species, the number of chemoreceptors can vary significantly. For example, the Escherichia coli laboratory strain K-12 has five chemoreceptors—Tsr, Tar, Trg, Tap, and Aer (2)—whereas many extraintestinal pathogenic E. coli strains lost either Trg or Tap or both, and several strains acquired additional chemoreceptors via a horizontal gene transfer (35). The number of chemoreceptors in P. aeruginosa strains varies from 21 to 32 with the average being 26. In this study, we focused on the evolution of three amino acid-sensing chemoreceptors—PctA, PctB, and PctC. Our results show that despite a very short timeline of divergence, active evolutionary changes occurred both at the genomic level (gene duplication, loss, and rearrangements) and at the level of individual amino acids, specifically in sensory domains. Comparative genome analysis revealed that gene duplication is a major driving force in the evolution of this chemoreceptor family: more than 60% of genomes analyzed in this study contained paralogous chemoreceptors of this type. Many paralogs originated from multiple independent gene duplication events. Possible fates for duplicated genes include neofunctionalization, subfunctionalization, or loss. We observed a substantial number of pseudogenes and a complete loss of *pctA*, *pctB*, and *pctC* genes (in various combinations) in several strains indicating “unsuccessful” evolutionary “experiments.” On the other hand, the birth of PctC in the common ancestor of the genus *Pseudomonas* and the birth of PctB in the common ancestor of Pseudomonas aeruginosa are examples of “successful” gene duplications. All “successful” duplication events resulted in very specific subfunctionalization of paralogs—substantial changes in the chemoreceptor sensory domain leading to changes in the ligand repertoire.

The difference between the sensory and signaling domains in terms of evolution is striking. The signaling domain remains essentially intact upon gene duplication—this was observed not only in the case of PctC and PctB duplication events but across all analyzed homologous subgroups. The signaling domain is under a strict selection pressure to maintain complex interactions between chemoreceptor homodimers, trimers-of-dimers, and interactions with other components of the chemotaxis machinery. Even a modest single amino acid substitution in the signaling domain often leads to a dramatic loss in fitness (36–38). Despite the presence of four homologous chemosensory pathways in P. aeruginosa and the fact that only a few amino acid residues in the signaling domain determine the pathway specificity (39), we found no evidence for changes that might lead to chemoreceptor switching from one pathway to another due to gene duplication.

In contrast, the sensory domain of chemoreceptor paralogs undergoes substantial changes. While its key functional determinant—a motif involved in binding the amino and carboxyl groups of amino acid ligands—remains intact, positions in the flanking regions are specifically conserved within but not between paralog clusters. Amino acid residues that are conserved across all clusters surround a ligand from four sides and impose strong binding constraints. Cluster-specific conserved residues establish additional interactions and determine the orientation of the ligand. These interactions also facilitate conformational changes upon ligand binding that would likely enable downstream signal transduction.

One of the questions that motivated our study was which of the three amino acid binding chemoreceptors in P. aeruginosa is ancestral. Results of our phylogenetic analysis showed that the multi-amino-acid-sensing PctA chemoreceptor was the ancestral one. Interestingly, multi-amino-acid-sensing dCache_1 domain-containing chemoreceptors were identified in other pseudomonads (19, 22) as well as in model organisms from other phyla, such as Bacillus subtilis (12), Sinorhizobium meliloti (40), or Vibrio cholerae (23). In addition, dCache-containing amino acid chemoreceptors with a restricted ligand range have been detected in B. subtilis (41), Campylobacter jejuni (26), and Pseudomonas syringae (20). Whether the evolutionary history of these receptors is similar to that reported remains to be determined.

So far, two sensor domains have been shown to be composed of two structural modules, namely, the dCache and the HBM domain (42), the latter comprising two stacked 4-helix bundle modules. In McpS, an HBM domain-containing chemoreceptor, chemoattractants bind to both modules, causing additive chemotactic responses (43). This finding raises the question whether a similar mechanism applies to dCache domains. We show that PctABC ligands bind to the membrane-distal module, and *in silico* docking studies did not provide any evidence for ligand binding at the membrane-proximal module, a finding that is in agreement with the large majority of structural information available for dCache domains (8, 16, 33, 44–48). So far, there is only a single dCache domain (TlpC receptor of Helicobacter pylori) that showed bound chemoeffector (lactate) in the membrane-proximal module (49). However, lactate binding to TlpC-LBD occurs with an affinity that is well below that observed for other dCache domains (8, 14–16, 19, 29). In addition, several reports suggest that dCache LBD-containing chemoreceptors are stimulated by the binding of ligand-binding proteins (12, 50). Taken together, data indicate that the large majority of dCache domains do not recognize ligands at their membrane-proximal module, and the function of this module might be to relay the ligand-induced conformational changes to the signaling domain via the second transmembrane helix or to recognize ligand loaded binding proteins.

P. aeruginosa is an opportunistic pathogen, which infects patients with a compromised immune system, burns, or cystic fibrosis (CF). A recent study revealed that a Δ*pctABC* triple mutant had a significant reduction in chemotaxis and immobilization along wounds of human CF airway epithelial cells (51), thus demonstrating the potential importance of amino acid-sensing chemoreceptors for infection. The birth of the PctB chemoreceptor might have been especially beneficial for efficient host colonization by P. aeruginosa. It enabled the pathogen to move toward increasing concentrations of glutamine, which is the most abundant amino acid in human cells (52). Another recent study (53) showed downregulation of PctA, PctB, and the aerotaxis receptor PA1561 in P. aeruginosa isolated from the sputum of CF patients. Our analysis shows that many P. aeruginosa strains isolated from sputum of CF patients lack one or two genes encoding the amino acid-sensing chemoreceptors. Perhaps, once the infection is established, chemotaxis might no longer be a critical feature for P. aeruginosa, and it appears that, similarly to other pathogens, chemotaxis is important primarily for the initial steps of infection and cell attachment. Chemotaxis to amino acids might be especially important in P. aeruginosa infection, because the amino acid concentration in sputum of patients with CF is high and it correlates with pulmonary disease severity (54).

## MATERIALS AND METHODS

### Data sources and bioinformatics software.

BLAST searches were carried out against a locally stored copy of the NCBI nonredundant bacterial database (18 March 2016). Gene neighborhoods were identified using the MiST 3.0 database (available at https://mistdb.com). Taxonomy information for phylogenetic profiling was retrieved from the Genome Taxonomy Database (30). Multiple sequence alignments using full-length protein sequences were constructed using the MAFFT (v7.310) L-INS-I algorithm (55). Alignments were edited in Jalview (56). The maximum likelihood tree was built in MEGA (57) using the JTT substitution model. Sequence logos were generated using WebLogo (58).

### Identification of homologs, phylogenetic profiling, and sequence conservation patterns.

Protein sequences of PctA homologs were retrieved using BLAST searches with P. aeruginosa PAO1 PctA, PctB, and PctC sequences as queries, with an E value threshold of ≤0.001. Hits with query coverage of ≥90% and sequence identity of ≥30% were collected, and a resulting data set contained 5,200 sequences. Clustering and redundancy reduction were performed using the following procedure: (i) run BLAST all-versus-all; (ii) collect best high-scoring pairs for each hit; (iii) collect best hits for each genome; (iv) reduce redundancy by clustering genomes if (a) the number of PctA, PctB, and PctC homologs is the same, (b) a query coverage of each blast hit equals 100%, and (c) sequence identity for all PctA, PctB, and PctC homologs for a given pair is 100%; and (v) from each cluster, pick representative sequences from a genome with the highest level of assembly. The resulting representative data set contained 2,143 sequences from the final set of genomes. Sequences were then aligned, and a maximum likelihood tree was built from this alignment. In order to reveal sequence conservation patterns within the dCache_1 domain, branches on the tree were compressed into clusters and sequence logos were generated based on the multiple sequence alignment of the dCache_1 regions within each cluster. A genome tree for phylogenetic profiling was prepared using the Genome Taxonomy Database (30). The number of PctA homologs in each of the collected genomes was determined using a custom script.

### Protein expression, purification, and microcalorimetric analyses.

Experiments for protein expression, purification, and microcalorimetric analysis were conducted as described in reference 29.

### Crystallization and data collection.

The three LBDs at 25 mg/ml were incubated with 3 mM ligand for 30 min on ice, and the excess of ligand was removed by buffer exchange using 10-kDa-cutoff filters (Amicon). The resulting proteins as well as the apo form were used for initial crystallization screening at 293 and 277 K, using the counterdiffusion technique (59), 0.1-mm-inner-diameter capillaries, and the CSK-24, AS-49, and PEG448-49 screening kits (Triana S&T, Granada, Spain). Crystal size improvement was achieved by increasing the capillary diameter to 0.2 mm as described in reference 60. Crystallization conditions are provided in Table S3 in the supplemental material. Crystallization trials with PctA-LBD were conducted with a large number of amino acids, but crystals appeared only with l-Ile, l-Trp, and l-Met. Attempts to crystallize the apo forms of the three proteins were unsuccessful. Crystals were subjected to cryoprotection by the addition of 15% (vol/vol) glycerol to the mother liquid followed by an overnight equilibration of the crystal-containing capillaries. The best-diffracting crystals were used to collect X-ray data diffraction at beamlines Promixa-I of SOLEIL (Paris, France), XALOC of ALBA (Barcelona, Spain), and ID23-1, ID23-2, ID29, and Bm14U of ESRF (Grenoble, France) synchrotron sources. Data were indexed and integrated with XDS (61), and the intensities were scaled and merged with Scala (62) of the CCP4 program suite (63).

10.1128/mBio.03066-19.9 TABLE S3 Conditions used for protein crystallization. Download Table S3, PDF file, 0.1 MB. Copyright © 2020 Gavira et al. 2020 Gavira et al. This content is distributed under the terms of the Creative Commons Attribution 4.0 International license.

### Structure determination and analysis.

Coordinates from the LBD of an uncharacterized chemoreceptor of Vibrio cholerae (PDB ID 3C8C, chain A truncated to polyalanine) were used as a search model for the molecular replacement of PctA-LBD-Ile X-ray data using Phaser (64). Refinement was initiated with phenix.refine (65) of the PHENIX suite (66) followed by automatic chain mutation in Coot (67). Consecutive cycles of simulated annealing, manual building, and water inspection were done prior to ligand positioning. The final refinement of ligand coordinates, B-factors, and occupancies was achieved following several cycles of refinement, including titration-libration-screw (TLS) parameterization. The final model was verified with Procheck (68) and MolProbity (69). A similar procedure was followed to solve the structures of PctA-LBD with l-Trp and l-Met but using the refined PctA-LBD as the search model. PctB-LBD-Arg and PctC-LBD-GABA were also solved using PctA-LBD as the initial search model, but in the case of PctC-LBD-GABA, a solution was found only when the anomalous contribution was included and used within the Rickshaw pipeline (70). Table 1 summarizes crystallographic data statistics and final model characteristics. All bound ligands were modeled at 100% occupancy. Secondary structural elements were determined with DSSP (71), and graphics were prepared with PyMOL (72). Structural comparison was done with the DALI server (73).

**TABLE 1:** Data collection and refinement statistics^*a*^

Statistic	Protein and ligand (PDB identifier)					
	PctA-LBD			PctB-LBD		PctC-LBD
	l-Ile (5T65)	l-Trp (5T7M)	l-Met (5LTX)	l-Gln (5LTO)	l-Arg (5LT9)	GABA (5LTV)
Data collection						
Beamline	Proxima 1 (SOLEIL)	ID23-1 (ESRF)	ID23-2 (ESRF)	Bm14U (ESRF)	ID23-1 (ESRF)	ID23-2 (ESRF)
Space group	P 6_1_	P 2_1_ 2_1_ 2_1_	P 2_1_ 2_1_ 2_1_	P 3_2_ 2 1	P 3_2_ 2 1	P 6_4_
Cell dimensions *a*, *b*, *c* (Å)	*a* = *b* = 132.61, 77.09	72.11, 78.41, 116.45	71.68, 76.56, 116.50	*a* = *b* = 111.43, 117.72	*a* = *b* = 111.73, 117.55	*a* = *b* = 209.77, 68.88
Chains in ASU	2	2	2	2	2	7
Resolution (Å)	33.15–2.20 (2.28–2.20)	58.23–2.25 (2.33–2.25)	46.36–2.02 (2.09–2.02)	74.63–3.46 (3.58–3.46)	36.32–3.00 (3.11–3.00)	48.63–2.31 (2.39–2.31)
*R*_merge_ (%)	6.1 (53.80)	13.34 (97.72)	8.0 (89.48)	18.03 (89.10)	11.44 (110.1)	16.12 (108.1)
*I*/σ*_I_*	20.99 (3.32)	10.84 (2.10)	12.65 (1.67)	14.21 (3.00)	13.76 (1.35)	9.49 (1.78)
Completeness (%)	99.96 (100.00)	99.94 (99.87)	100.00 (99.00)	100.00 (100.00)	100.0 (100.0)	100.0 (100.0)
Unique reflections, no.	39,288 (3,907)	32,012 (3,140)	42,574 (4,160)	11,402 (1,118)	17,399 (1,726)	76,066 (7,552)
Multiplicity	7.0 (7.0)	8.2 (8.5)	4.8 (4.8)	9.6 (9.9)	9.0 (6.1)	7.0 (6.7)
CC(1/2)	0.999 (0.866)	0.996 (0.791)	0.999 (0.682)	0.995 (0.813)	0.998 (0.603)	0.996 (0.547)

Refinement						
Resolution (Å)	47.9–2.2	46.7–1.8	46.36–2.02	74.63–3.46	36.32–3.0	48.63–2.31
*R*_work_/*R*_free_ (%)	16.63/20.38	18.52/23.40	18.80/21.84	21.73/27.73	21.56/24.97	17.72/22.55
No. of atoms	4,163	4,019	4,438	3,760	3,846	12,780
Protein	3,902	3,787	4,026	3,729	3,834	11,913
Ligands	13	13	12	6	7	26
Water	248	219	374	13	6	704
B-factor, all atoms (Å^2^)	48.4	27.9	43.88	77.05	86.44	45.33
RMS deviations						
Bond lengths (Å)	0.009	0.011	0.006	0.003	0.005	0.012
Bond angles (°)	1.14	1.52	0.75	0.58	0.71	1.22
Ramachandran (%)						
Favored	99	99	98	95	97	99
Outliers	0.2	0	0	0	0.41	0

a Values in parentheses are for highest-resolution shell. ASU, asymmetric unit; RMS, root mean square.

### Molecular dynamics simulations.

All protein-ligand complexes were subjected to structural refinement with the *profix* program from the JACKAL package (74). The hydrogen atoms were added to the titratable groups based on the relationship between neutral pH and pK_a_ calculated with PROPKA software (75) using Charmm22 force field parameters in VMD software (76). To avoid initial structural clashes, all protein-ligand complexes were first relaxed using NAMD software (77) for 20,000 steps via a conjugate gradient algorithm. The minimized structures were then subjected to equilibration at 298 K for 1 ns using 2-fs time steps and molecular dynamic simulations for another 10 ns, at a constant temperature of 298 K. MD simulations were done using the GB implicit solvent model, applying dielectric constants 80 and 1 to the solvent and solute, respectively. In order to mimic the movement restriction of the proteins due to their interactions with membrane and to keep the consistency between proteins, the Cα atoms of Leu42 and Ile262 in PctA, which correspond to Leu42 and Val262 in PctB and Thr42 and Leu265 in PctC, were fixed during MD simulations. Each 0.2 ns, the snapshot of the complex was exported to the DCD trajectory file for further structural analysis. The visual examination of the MD trajectories of proteins with bound ligands showed that, although in most of the cases both monomers of the homodimers retained their ligands, in three cases, PctA_ILE, PctB_GLN, and PctC_GABA, one of the ligands was released from the binding pocket. The probability of residues to establish hydrogen bonds was calculated with the VMD software (76) using standard criteria, namely, a donor-acceptor distance less than 3 Å and the donor-hydrogen-acceptor angle of at least 20°. The probability of hydrophobic interactions between residues was calculated by an in-house algorithm based on the distance of less than 4 Å.

### *In silico* docking experiments.

The atomic structures of PctA-LBD, PctB-LBD, and PctC-LBD were refined and optimized with the Protein Preparation Wizard of the Schrödinger suite (78). The coordinates of amino acids and GABA and the Natural Compounds Collection were obtained from the Zinc database (79) and optimized by LigPrep (LigPrep, version 2.5, 2012; Schrödinger, LLC, New York, NY). The binding energy values from the virtual docking of the amino acids and GABA and the virtual screening of the Natural Compounds collection were obtained from a simulation using the Glide Dock XP mode (34).

### Data availability.

The coordinates and the experimental structure factors have been deposited in the Protein Data Bank with the following identifiers: l-Ile, 5T65; l-Trp, 5T7M; l-Met, 5LTX; l-Gln, 5LTO; l-Arg, 5LT9; GABA, 5LTV.

## References

[1] Wuichet K, Zhulin IB 2010 Origins and diversification of a complex signal transduction system in prokaryotes. Sci Signal 3:ra50. doi:10.1126/scisignal.2000724. 20587806 PMC3401578

[2] Parkinson JS, Hazelbauer GL, Falke JJ 2015 Signaling and sensory adaptation in Escherichia coli chemoreceptors: 2015 update. Trends Microbiol 23:257–266. doi:10.1016/j.tim.2015.03.003. 25834953 PMC4417406

[3] Hazelbauer GL, Falke JJ, Parkinson JS 2008 Bacterial chemoreceptors: high-performance signaling in networked arrays. Trends Biochem Sci 33:9–19. doi:10.1016/j.tibs.2007.09.014. 18165013 PMC2890293

[4] Sampedro I, Parales RE, Krell T, Hill JE 2015 Pseudomonas chemotaxis. FEMS Microbiol Rev 39:17–46. doi:10.1111/1574-6976.12081. 25100612

[5] Ortega A, Zhulin IB, Krell T 2017 Sensory repertoire of bacterial chemoreceptors. Microbiol Mol Biol Rev 81:e00033-17. doi:10.1128/MMBR.00033-17. PMC5706747 29070658

[6] Alexandre G, Greer-Phillips S, Zhulin IB 2004 Ecological role of energy taxis in microorganisms. FEMS Microbiol Rev 28:113–126. doi:10.1016/j.femsre.2003.10.003. 14975533

[7] Scharf BE, Hynes MF, Alexandre GM 2016 Chemotaxis signaling systems in model beneficial plant-bacteria associations. Plant Mol Biol 90:549–559. doi:10.1007/s11103-016-0432-4. 26797793

[8] Corral-Lugo A, Matilla MA, Martin-Mora D, Silva Jimenez H, Mesa Torres N, Kato J, Hida A, Oku S, Conejero-Muriel M, Gavira JA, Krell T 2018 High-affinity chemotaxis to histamine mediated by the TlpQ chemoreceptor of the human pathogen *Pseudomonas aeruginosa*. mBio 9:e01894-18. doi:10.1128/mBio.01894-18. 30425146 PMC6234866

[9] Martin-Mora D, Ortega A, Matilla MA, Martinez-Rodriguez S, Gavira JA, Krell T 2019 The molecular mechanism of nitrate chemotaxis via direct ligand binding to the PilJ domain of McpN. mBio 10:e02334-18. doi:10.1128/mBio.02334-18. 30782655 PMC6381276

[10] Upadhyay AA, Fleetwood AD, Adebali O, Finn RD, Zhulin IB 2016 Cache domains that are homologous to, but different from PAS domains comprise the largest superfamily of extracellular sensors in prokaryotes. PLoS Comput Biol 12:e1004862. doi:10.1371/journal.pcbi.1004862. 27049771 PMC4822843

[11] Hothorn M, Dabi T, Chory J 2011 Structural basis for cytokinin recognition by *Arabidopsis thaliana* histidine kinase 4. Nat Chem Biol 7:766–768. doi:10.1038/nchembio.667. 21964459 PMC3197759

[12] Glekas GD, Mulhern BJ, Kroc A, Duelfer KA, Lei V, Rao CV, Ordal GW 2012 The *Bacillus subtilis* chemoreceptor McpC senses multiple ligands using two discrete mechanisms. J Biol Chem 287:39412–39418. doi:10.1074/jbc.M112.413518. 23038252 PMC3501012

[13] Reyes-Darias JA, García V, Rico-Jiménez M, Corral-Lugo A, Lesouhaitier O, Juárez-Hernández D, Yang Y, Bi S, Feuilloley M, Muñoz-Rojas J, Sourjik V, Krell T 2015 Specific gamma-aminobutyrate chemotaxis in pseudomonads with different lifestyle. Mol Microbiol 97:488–501. doi:10.1111/mmi.13045. 25921834

[14] Webb BA, Karl Compton K, Castaneda Saldana R, Arapov TD, Keith Ray W, Helm RF, Scharf BE 2017 *Sinorhizobium meliloti* chemotaxis to quaternary ammonium compounds is mediated by the chemoreceptor McpX. Mol Microbiol 103:333–346. doi:10.1111/mmi.13561. 27748981

[15] Fernandez M, Morel B, Corral-Lugo A, Krell T 2016 Identification of a chemoreceptor that specifically mediates chemotaxis toward metabolizable purine derivatives. Mol Microbiol 99:34–42. doi:10.1111/mmi.13215. 26355499

[16] Nishiyama S, Takahashi Y, Yamamoto K, Suzuki D, Itoh Y, Sumita K, Uchida Y, Homma M, Imada K, Kawagishi I 2016 Identification of a *Vibrio cholerae* chemoreceptor that senses taurine and amino acids as attractants. Sci Rep 6:20866. doi:10.1038/srep20866. 26878914 PMC4754685

[17] Feng H, Zhang N, Du W, Zhang H, Liu Y, Fu R, Shao J, Zhang G, Shen QR, Zhang R 2018 Identification of chemotaxis compounds in root exudates and their sensing chemoreceptors in plant growth-promoting rhizobacteria *Bacillus amyloliquefaciens* SQR9. Mol Plant Microbe Interact 31:995–1005. doi:10.1094/MPMI-01-18-0003-R. 29714096

[18] Reyes-Darias JA, Yang Y, Sourjik V, Krell T 2015 Correlation between signal input and output in PctA and PctB amino acid chemoreceptor of *Pseudomonas aeruginosa*. Mol Microbiol 96:513–525. doi:10.1111/mmi.12953. 25641105

[19] Corral-Lugo A, De la Torre J, Matilla MA, Fernández M, Morel B, Espinosa-Urgel M, Krell T 2016 Assessment of the contribution of chemoreceptor-based signaling to biofilm formation. Environ Microbiol 18:3355–3372. doi:10.1111/1462-2920.13170. 26662997

[20] McKellar JL, Minnell JJ, Gerth ML 2015 A high-throughput screen for ligand binding reveals the specificities of three amino acid chemoreceptors from *Pseudomonas syringae* pv. *actinidiae*. Mol Microbiol 96:694–707. doi:10.1111/mmi.12964. 25656450

[21] Taguchi K, Fukutomi H, Kuroda A, Kato J, Ohtake H 1997 Genetic identification of chemotactic transducers for amino acids in *Pseudomonas aeruginosa*. Microbiology 143:3223–3229. doi:10.1099/00221287-143-10-3223. 9353923

[22] Oku S, Komatsu A, Tajima T, Nakashimada Y, Kato J 2012 Identification of chemotaxis sensory proteins for amino acids in *Pseudomonas fluorescens* Pf0-1 and their involvement in chemotaxis to tomato root exudate and root colonization. Microbes Environ 27:462–469. doi:10.1264/jsme2.me12005. 22972385 PMC4103555

[23] Nishiyama S, Suzuki D, Itoh Y, Suzuki K, Tajima H, Hyakutake A, Homma M, Butler-Wu SM, Camilli A, Kawagishi I 2012 Mlp24 (McpX) of *Vibrio cholerae* implicated in pathogenicity functions as a chemoreceptor for multiple amino acids. Infect Immun 80:3170–3178. doi:10.1128/IAI.00039-12. 22753378 PMC3418727

[24] Brennan CA, DeLoney-Marino CR, Mandel MJ 2013 Chemoreceptor VfcA mediates amino acid chemotaxis in *Vibrio fischeri*. Appl Environ Microbiol 79:1889–1896. doi:10.1128/AEM.03794-12. 23315744 PMC3592237

[25] Cerda O, Rivas A, Toledo H 2003 *Helicobacter pylori* strain ATCC700392 encodes a methyl-accepting chemotaxis receptor protein (MCP) for arginine and sodium bicarbonate. FEMS Microbiol Lett 224:175–181. doi:10.1016/S0378-1097(03)00423-3. 12892880

[26] Hartley-Tassell LE, Shewell LK, Day CJ, Wilson JC, Sandhu R, Ketley JM, Korolik V 2010 Identification and characterization of the aspartate chemosensory receptor of *Campylobacter jejuni*. Mol Microbiol 75:710–730. doi:10.1111/j.1365-2958.2009.07010.x. 20025667

[27] Kokoeva MV, Oesterhelt D 2000 BasT, a membrane-bound transducer protein for amino acid detection in *Halobacterium salinarum*. Mol Microbiol 35:647–656. doi:10.1046/j.1365-2958.2000.01735.x. 10672186

[28] Kuroda A, Kumano T, Taguchi K, Nikata T, Kato J, Ohtake H 1995 Molecular cloning and characterization of a chemotactic transducer gene in *Pseudomonas aeruginosa*. J Bacteriol 177:7019–7025. doi:10.1128/jb.177.24.7019-7025.1995. 8522505 PMC177577

[29] Rico-Jimenez M, Munoz-Martinez F, Garcia-Fontana C, Fernandez M, Morel B, Ortega A, Ramos JL, Krell T 2013 Paralogous chemoreceptors mediate chemotaxis towards protein amino acids and the non-protein amino acid gamma-aminobutyrate (GABA). Mol Microbiol 88:1230–1243. doi:10.1111/mmi.12255. 23650915

[30] Parks DH, Chuvochina M, Waite DW, Rinke C, Skarshewski A, Chaumeil PA, Hugenholtz P 2018 A standardized bacterial taxonomy based on genome phylogeny substantially revises the tree of life. Nat Biotechnol 36:996–1004. doi:10.1038/nbt.4229. 30148503

[31] Ottemann KM, Xiao W, Shin YK, Koshland DE Jr. 1999 A piston model for transmembrane signaling of the aspartate receptor. Science 285:1751–1754. doi:10.1126/science.285.5434.1751. 10481014

[32] Yu D, Ma X, Tu Y, Lai L 2015 Both piston-like and rotational motions are present in bacterial chemoreceptor signaling. Sci Rep 5:8640. doi:10.1038/srep08640. 25728261 PMC4345343

[33] Gavira JA, Ortega A, Martin-Mora D, Conejero-Muriel MT, Corral-Lugo A, Morel B, Matilla MA, Krell T 2018 Structural basis for polyamine binding at the dCACHE domain of the McpU chemoreceptor from *Pseudomonas putida*. J Mol Biol 430:1950–1963. doi:10.1016/j.jmb.2018.05.008. 29758259

[34] Friesner RA, Murphy RB, Repasky MP, Frye LL, Greenwood JR, Halgren TA, Sanschagrin PC, Mainz DT 2006 Extra precision glide: docking and scoring incorporating a model of hydrophobic enclosure for protein-ligand complexes. J Med Chem 49:6177–6196. doi:10.1021/jm051256o. 17034125

[35] Borziak K, Fleetwood AD, Zhulin IB 2013 Chemoreceptor gene loss and acquisition via horizontal gene transfer in *Escherichia coli*. J Bacteriol 195:3596–3602. doi:10.1128/JB.00421-13. 23749975 PMC3754581

[36] Gosink KK, Zhao Y, Parkinson JS 2011 Mutational analysis of N381, a key trimer contact residue in Tsr, the *Escherichia coli* serine chemoreceptor. J Bacteriol 193:6452–6460. doi:10.1128/JB.05887-11. 21965562 PMC3232890

[37] Ortega DR, Yang C, Ames P, Baudry J, Parkinson JS, Zhulin IB 2013 A phenylalanine rotameric switch for signal-state control in bacterial chemoreceptors. Nat Commun 4:2881. doi:10.1038/ncomms3881. 24335957 PMC4310728

[38] Lai RZ, Gosink KK, Parkinson JS 2017 Signaling consequences of structural lesions that alter the stability of chemoreceptor trimers of dimers. J Mol Biol 429:823–835. doi:10.1016/j.jmb.2017.02.007. 28215934 PMC5370551

[39] Ortega DR, Fleetwood AD, Krell T, Harwood CS, Jensen GJ, Zhulin IB 2017 Assigning chemoreceptors to chemosensory pathways in *Pseudomonas aeruginosa*. Proc Natl Acad Sci U S A 114:12809–12814. doi:10.1073/pnas.1708842114. 29133402 PMC5715753

[40] Webb BA, Compton KK, Del Campo JSM, Taylor D, Sobrado P, Scharf BE 2017 *Sinorhizobium meliloti* chemotaxis to multiple amino acids is mediated by the chemoreceptor McpU. Mol Plant Microbe Interact 30:770–777. doi:10.1094/MPMI-04-17-0096-R. 28745538

[41] Glekas GD, Foster RM, Cates JR, Estrella JA, Wawrzyniak MJ, Rao CV, Ordal GW 2010 A PAS domain binds asparagine in the chemotaxis receptor McpB in *Bacillus subtilis*. J Biol Chem 285:1870–1878. doi:10.1074/jbc.M109.072108. 19864420 PMC2804345

[42] Ortega A, Krell T 2014 The HBM domain: introducing bimodularity to bacterial sensing. Protein Sci 23:332–336. doi:10.1002/pro.2410. 24347303 PMC3945841

[43] Pineda-Molina E, Reyes-Darias J-A, Lacal J, Ramos JL, García-Ruiz JM, Gavira JA, Krell T 2012 Evidence for chemoreceptors with bimodular ligand-binding regions harboring two signal-binding sites. Proc Natl Acad Sci U S A 109:18926–18931. doi:10.1073/pnas.1201400109. 23112148 PMC3503224

[44] Liu YC, Machuca MA, Beckham SA, Gunzburg MJ, Roujeinikova A 2015 Structural basis for amino-acid recognition and transmembrane signalling by tandem Per-Arnt-Sim (tandem PAS) chemoreceptor sensory domains. Acta Crystallogr D Biol Crystallogr 71:2127–2136. doi:10.1107/S139900471501384X. 26457436

[45] Takahashi Y, Nishiyama S-I, Sumita K, Kawagishi I, Imada K, Takahashi Y, Nishiyama S-I, Sumita K, Kawagishi I, Imada K 2019 Calcium ions modulate amino acid sensing of the chemoreceptor Mlp24 of *Vibrio cholerae*. J Bacteriol 201:e00779-18. doi:10.1128/JB.00779-18. 30745373 PMC6456857

[46] Shrestha M, Compton KK, Mancl JM, Webb BA, Brown AM, Scharf BE, Schubot FD 2018 Structure of the sensory domain of McpX from *Sinorhizobium meliloti*, the first known bacterial chemotactic sensor for quaternary ammonium compounds. Biochem J 475:3949–3962. doi:10.1042/BCJ20180769. 30442721

[47] Wu R, Gu M, Wilton R, Babnigg G, Kim Y, Pokkuluri PR, Szurmant H, Joachimiak A, Schiffer M 2013 Insight into the sporulation phosphorelay: crystal structure of the sensor domain of *Bacillus subtilis* histidine kinase, KinD. Protein Sci 22:564–576. doi:10.1002/pro.2237. 23436677 PMC3649258

[48] Cheung J, Hendrickson WA 2008 Crystal structures of C4-dicarboxylate ligand complexes with sensor domains of histidine kinases DcuS and DctB. J Biol Chem 283:30256–30265. doi:10.1074/jbc.M805253200. 18701447 PMC2573060

[49] Machuca MA, Johnson KS, Liu YC, Steer DL, Ottemann KM, Roujeinikova A 2017 *Helicobacter pylori* chemoreceptor TlpC mediates chemotaxis to lactate. Sci Rep 7:14089. doi:10.1038/s41598-017-14372-2. 29075010 PMC5658362

[50] Machuca MA, Liu YC, Beckham SA, Gunzburg MJ, Roujeinikova A 2016 The crystal structure of the tandem-PAS sensing domain of *Campylobacter jejuni* chemoreceptor Tlp1 suggests indirect mechanism of ligand recognition. J Struct Biol 194:205–213. doi:10.1016/j.jsb.2016.02.019. 26923153

[51] Schwarzer C, Fischer H, Machen TE 2016 Chemotaxis and binding of *Pseudomonas aeruginosa* to scratch-wounded human cystic fibrosis airway epithelial cells. PLoS One 11:e0150109. doi:10.1371/journal.pone.0150109. 27031335 PMC4816407

[52] Brosnan JT 2003 Interorgan amino acid transport and its regulation. J Nutr 133:2068S–2072S. doi:10.1093/jn/133.6.2068S. 12771367

[53] Kamath KS, Pascovici D, Penesyan A, Goel A, Venkatakrishnan V, Paulsen IT, Packer NH, Molloy MP 2016 *Pseudomonas aeruginosa* cell membrane protein expression from phenotypically diverse cystic fibrosis isolates demonstrates host-specific adaptations. J Proteome Res 15:2152–2163. doi:10.1021/acs.jproteome.6b00058. 27246823

[54] Thomas SR, Ray A, Hodson ME, Pitt TL 2000 Increased sputum amino acid concentrations and auxotrophy of *Pseudomonas aeruginosa* in severe cystic fibrosis lung disease. Thorax 55:795–797. doi:10.1136/thorax.55.9.795. 10950901 PMC1745865

[55] Katoh K, Standley DM 2013 MAFFT multiple sequence alignment software version 7: improvements in performance and usability. Mol Biol Evol 30:772–780. doi:10.1093/molbev/mst010. 23329690 PMC3603318

[56] Waterhouse AM, Procter JB, Martin DM, Clamp M, Barton GJ 2009 Jalview version 2—a multiple sequence alignment editor and analysis workbench. Bioinformatics 25:1189–1191. doi:10.1093/bioinformatics/btp033. 19151095 PMC2672624

[57] Tamura K, Stecher G, Peterson D, Filipski A, Kumar S 2013 MEGA6: Molecular Evolutionary Genetics Analysis version 6.0. Mol Biol Evol 30:2725–2729. doi:10.1093/molbev/mst197. 24132122 PMC3840312

[58] Crooks GE, Hon G, Chandonia JM, Brenner SE 2004 WebLogo: a sequence logo generator. Genome Res 14:1188–1190. doi:10.1101/gr.849004. 15173120 PMC419797

[59] Otalora F, Gavira JA, Ng JD, Garcia-Ruiz JM 2009 Counterdiffusion methods applied to protein crystallization. Prog Biophys Mol Biol 101:26–37. doi:10.1016/j.pbiomolbio.2009.12.004. 20018206

[60] Rico-Jiménez M, Muñoz-Martínez F, Krell T, Gavira JA, Pineda-Molina E 2013 Purification, crystallization and preliminary crystallographic analysis of the ligand-binding regions of the PctA and PctB chemoreceptors from *Pseudomonas aeruginosa* in complex with amino acids. Acta Crystallogr Sect F Struct Biol Cryst Commun 69:1431–1435. doi:10.1107/S1744309113023592. PMC3855737 24316847

[61] Kabsch W 2010 XDS. Acta Crystallogr D Biol Crystallogr 66:125–132. doi:10.1107/S0907444909047337. 20124692 PMC2815665

[62] Evans P 2006 Scaling and assessment of data quality. Acta Crystallogr D Biol Crystallogr 62:72–82. doi:10.1107/S0907444905036693. 16369096

[63] Collaborative Computational Project, Number 4. 1994 The CCP4 suite: programs for protein crystallography. Acta Crystallogr D Biol Crystallogr 50:760–763. doi:10.1107/S0907444994003112. 15299374

[64] McCoy AJ, Grosse-Kunstleve RW, Adams PD, Winn MD, Storoni LC, Read RJ 2007 Phaser crystallographic software. J Appl Crystallogr 40:658–674. doi:10.1107/S0021889807021206. 19461840 PMC2483472

[65] Afonine PV, Mustyakimov M, Grosse-Kunstleve RW, Moriarty NW, Langan P, Adams PD 2010 Joint X-ray and neutron refinement with phenix.refine. Acta Crystallogr D Biol Crystallogr 66:1153–1163. doi:10.1107/S0907444910026582. 21041930 PMC2967420

[66] Adams PD, Afonine PV, Bunkoczi G, Chen VB, Davis IW, Echols N, Headd JJ, Hung L-W, Kapral GJ, Grosse-Kunstleve RW, McCoy AJ, Moriarty NW, Oeffner R, Read RJ, Richardson DC, Richardson JS, Terwilliger TC, Zwart PH 2010 PHENIX: a comprehensive Python-based system for macromolecular structure solution. Acta Crystallogr D Biol Crystallogr 66:213–221. doi:10.1107/S0907444909052925. 20124702 PMC2815670

[67] Emsley P, Lohkamp B, Scott WG, Cowtan K 2010 Features and development of Coot. Acta Crystallogr D Biol Crystallogr 66:486–501. doi:10.1107/S0907444910007493. 20383002 PMC2852313

[68] Laskowski RA, MacArthur MW, Moss DS, Thornton JM 1993 PROCHECK: a program to check the stereochemical quality of protein structures. J Appl Crystallogr 26:283–291. doi:10.1107/S0021889892009944.

[69] Chen VB, Arendall WB III, Headd JJ, Keedy DA, Immormino RM, Kapral GJ, Murray LW, Richardson JS, Richardson DC 2010 MolProbity: all-atom structure validation for macromolecular crystallography. Acta Crystallogr D Biol Crystallogr 66:12–21. doi:10.1107/S0907444909042073. 20057044 PMC2803126

[70] Panjikar S, Parthasarathy V, Lamzin VS, Weiss MS, Tucker PA 2005 Auto-Rickshaw: an automated crystal structure determination platform as an efficient tool for the validation of an X-ray diffraction experiment. Acta Crystallogr D Biol Crystallogr 61:449–457. doi:10.1107/S0907444905001307. 15805600

[71] Kabsch W, Sander C 1983 Dictionary of protein secondary structure: pattern recognition of hydrogen-bonded and geometrical features. Biopolymers 22:2577–2637. doi:10.1002/bip.360221211. 6667333

[72] Schrodinger L 2010 The PyMOL molecular graphics system, version 1.3r1.

[73] Holm L, Park J 2000 DaliLite workbench for protein structure comparison. Bioinformatics 16:566–567. doi:10.1093/bioinformatics/16.6.566. 10980157

[74] Xiang JZ, Honig B 2002 JACKAL: a protein structure modeling package. Columbia University and Howard Hughes Medical Institute, New York, NY.

[75] Olsson MH, Sondergaard CR, Rostkowski M, Jensen JH 2011 PROPKA3: consistent treatment of internal and surface residues in empirical pKa predictions. J Chem Theory Comput 7:525–537. doi:10.1021/ct100578z. 26596171

[76] Humphrey W, Dalke A, Schulten K 1996 VMD: visual molecular dynamics. J Mol Graph 14:33–38. doi:10.1016/0263-7855(96)00018-5. 8744570

[77] Phillips JC, Braun R, Wang W, Gumbart J, Tajkhorshid E, Villa E, Chipot C, Skeel RD, Kale L, Schulten K 2005 Scalable molecular dynamics with NAMD. J Comput Chem 26:1781–1802. doi:10.1002/jcc.20289. 16222654 PMC2486339

[78] Sastry GM, Adzhigirey M, Day T, Annabhimoju R, Sherman W 2013 Protein and ligand preparation: parameters, protocols, and influence on virtual screening enrichments. J Comput Aided Mol Des 27:221–234. doi:10.1007/s10822-013-9644-8. 23579614

[79] Irwin JJ, Sterling T, Mysinger MM, Bolstad ES, Coleman RG 2012 ZINC: a free tool to discover chemistry for biology. J Chem Inf Model 52:1757–1768. doi:10.1021/ci3001277. 22587354 PMC3402020

[80] Laskowski RA, Swindells MB 2011 LigPlot+: multiple ligand-protein interaction diagrams for drug discovery. J Chem Inf Model 51:2778–2786. doi:10.1021/ci200227u. 21919503

