# Supplementary figures and images for: How Bacterial Chemoreceptors Evolve Novel Ligand Specificities
Source: mBio. 2020 Jan 21;11(1):e03066-19. doi: 10.1128/mBio.03066-19 (PMC6974571; doi:10.1128/mBio.03066-19)

Supp. Figures

Fig. S1

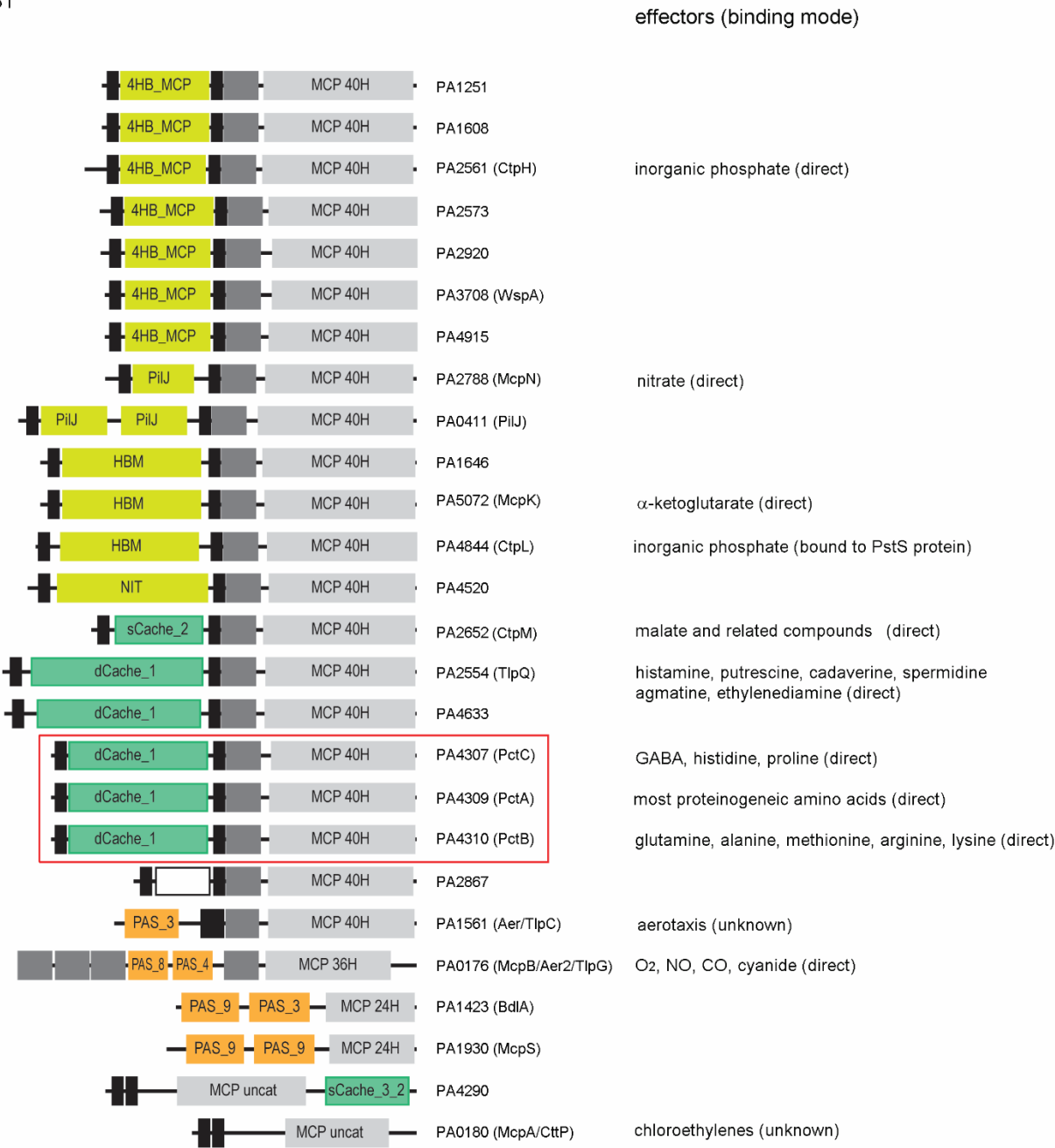

Supplement: FIG S1 [file mBio.03066-19-sf001.pdf]

Fig. S3

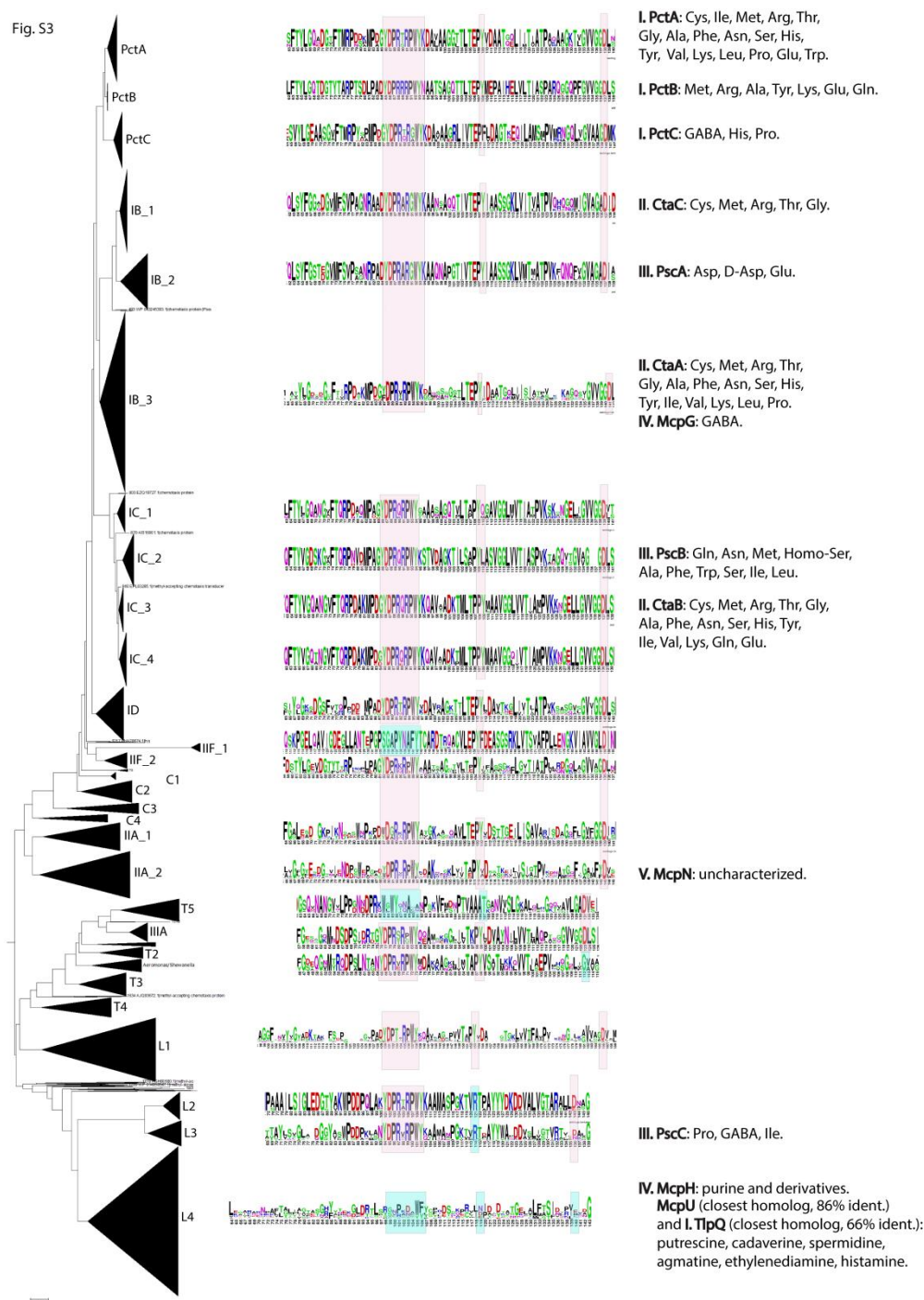

Supplement: FIG S3 [file mBio.03066-19-sf003.pdf]

Fig. S4

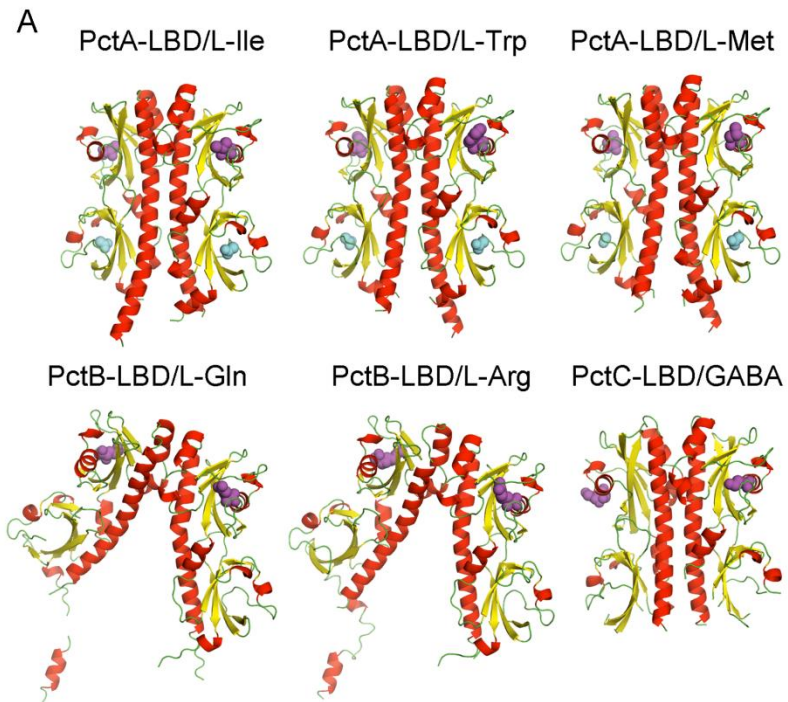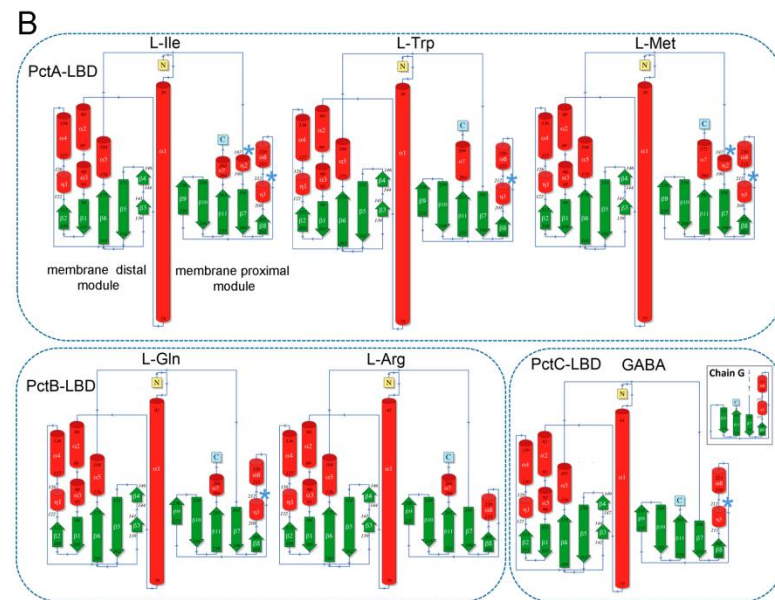

Supplement: FIG S4 [file mBio.03066-19-sf004.pdf]

Fig. S5

PctA-LBD/L-Trp

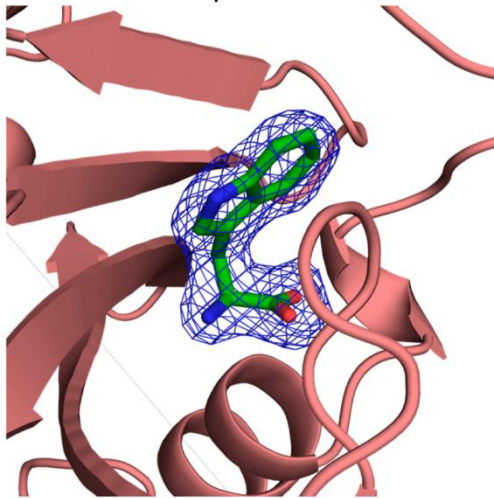

PctA-LBD/L-Ile

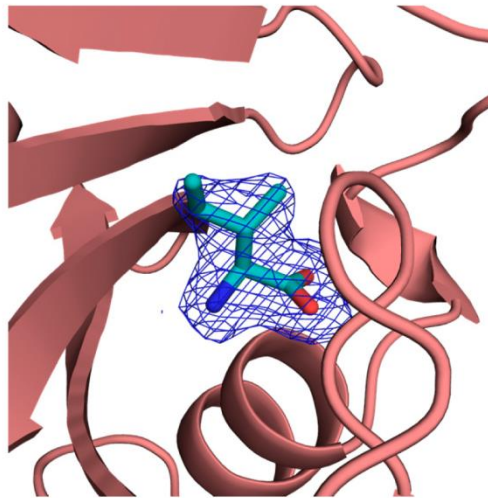

PctA-LBD/L-Met

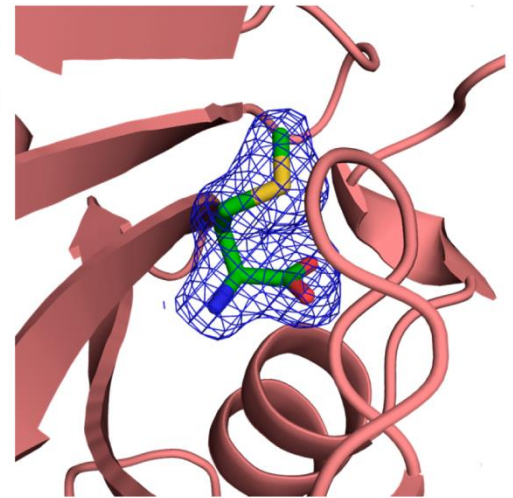

PctB-LBD/L-Gln

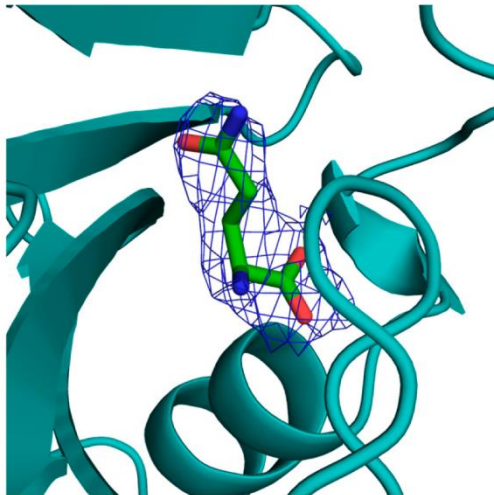

PctB-LBD/L-Arg

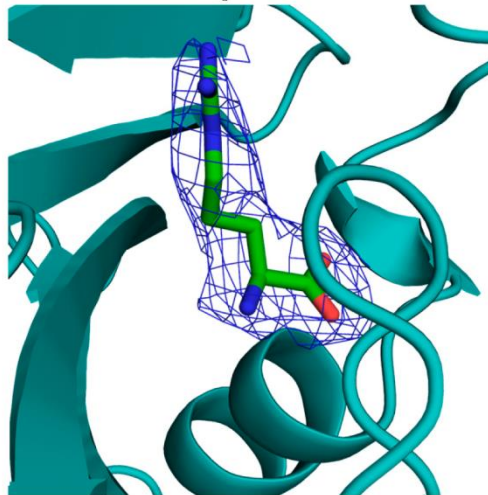

PctC-LBD/GABA

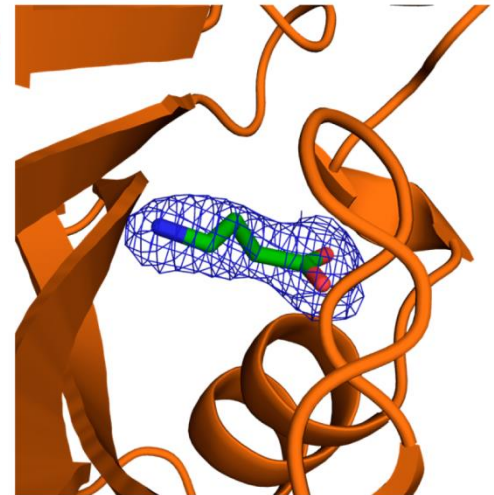

Supplement: FIG S5 [file mBio.03066-19-sf005.pdf]
