# Supplementary material for: How Bacterial Chemoreceptors Evolve Novel Ligand Specificities
Source: mBio. 2020 Jan 21;11(1):e03066-19. doi: 10.1128/mBio.03066-19 (PMC6974571; doi:10.1128/mBio.03066-19)
Supplement: FIG S2 [file mBio.03066-19-sf002.pdf]

Fig. S2

A

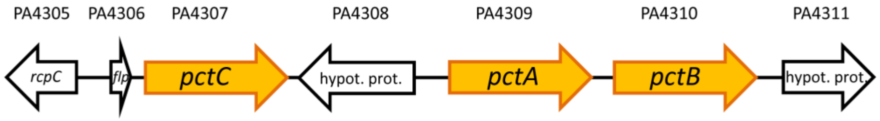

B

```
                10        20        30        40        50        60        70
                 |         |         |         |         |         |         |
PctA    MIKSLKFSHKILLAASLVVFAAFALFTLYNDYLQRNAIREDLESYLREMGDVTSSNIQNWLGGRLLLVEQ
PctB    MIKSLKFSHKILLAAALVVIATFSLFTLYNDSLQRASIREDLEDYLHEMGEITASNVQNWLSGRILLIEN
PctC    MLRSLSFAKKILLAAALVVVFAFSCFILYNDYRQREAVRTDTENYLGEIGTLTASNIQSWLEGRMHLVEG

                80        90       100       110       120       130       140
                 |         |         |         |         |         |         |
PctA    TAQTLARDHSP--ETVSALLEQPALTSTFSFTYLGQQD-GVFTMRPDSPMPAGYDPRSRPWYKDAVAAGG
PctB    LAQTLARDHSP--ETTQALLEQPLLGSTFLFTYLGQTD-GTYTARPTSDLPADYDPRRRPWYNAATSAGQ
PctC    LASQLALLDQPDEANIARQLEQPVFSRNFASVYLGEAASGTFTMRPYDAMPEGYDPRTRAWYKDALAADR

               150       160       170       180       190       200       210
                 |         |         |         |         |         |         |
PctA    LTLTEPYVDAATQELIITAATPVKAAGNTLGVVGGDLSLKTLVQIINSLDFSGMGYAFLVSGDGKILVHP
PctB    TTLTEPYMEPAIHELVLTIASPARQGGQPFGVVGGDLSLQTVVKIINSLDFGGMGYAFLVSGDGKILVHP
PctC    LIVTEPFVDAGTGEQILAMSLPVRHAGQLLGVAAGEWKLETLTAILNSLKFDGAGYAFLVSDAGKILLHP

               220       230       240       250       260       270       280
                 |         |         |         |         |         |         |
PctA    DKEQVMKTLSEVYPQNTPKIATGFSEAELHGHTRILAFTPIKGLPSVTWYLALSIDKDKAYAMLSKFRVS
PctB    DKDQVMKSLSDVYPRNTPKIGSGFSEAELHGNTRILSFSPVKGLSGLDWYIGISVDKDKAYAMLTKLRTS
PctC    DSGLVLKTLAEAYPKGAPNIVPGVHEVELDGSSQFVSFTPVKGLPGVTWYVALVLDRDTAYSMLSEFRTS

               290       300       310       320       330       340       350
                 |         |         |         |         |         |         |
PctA    AIAAALISIVAILVLLGLLIRLLMQPLHLMGRAMQDIAQGEGDLTKRLAVTSRDEFGVLGDAFNQFVERI
PctB    AIVAALIAVVAIVLLLGMLIRVLMQPLTDMGRAMQDIAQGEGDLTKRLKVTSNDEFGALAISFNRFVERI
PctC    AIVATLIAVVGIMLLLGMLIRVLMQPLTDMGRAMQDIAQGEGDLTKRLKVTSNDEFGTLANAFNRFVERI

               360       370       380       390       400       410       420
                 |         |         |         |         |         |         |
PctA    HRSIREVAGTAHKLHDVSQLVVNASNSSMANSDEQSNRTNSVAAAINELGAAAQEIARNAADASHHASDA
PctB    HESIREVAGTARQLHDVAQLVVNASNSSMANSDEQSNRTNSVAAAINELGAAAQEIARNAADASHHASDA
PctC    HESIREVAGTARQLHDVAQLVVNASNSSMANSDEQSNRTNSVAAAINELGAAAQEIARNAADASHHASDA

               430       440       450       460       470       480       490
                 |         |         |         |         |         |         |
PctA    NHQAEDGKQVVEQTIRAMNELSEKISASCANIEALNSRTVNIGQILEVIKGISEQTNLLALNAAIEAARA
PctB    NHQAEDGKQVVEQTIRAMNELSEKISASCANIEALNSRTVNIGQILEVIKGISEQTNLLALNAAIEAARA
PctC    NHQAEDGKQVVEQTIRAMNELSEKISASCANIEALNSRTVNIGQILEVIKGISEQTNLLALNAAIEAARD

               500       510       520       530       540       550       560
                 |         |         |         |         |         |         |
PctA    GEAGRGFAVVADEVRNLAHRAQESAQQIQKMIEELQVGAREAVATMTESQRYSLESVEIANRAGESLSSV
PctB    GEAGRGFAVVADEVRNLAHRAQESAQQIQKMIEELQIGAQEAVSTMTESQRYSLESVEIANRAGERLSSV
PctC    GEAGRGFAVVADEVRNLAHRAQESAQQIQKMIEELQVGAREAVATMTESQRYSLESVEIANRAGERLGSV

               570       580       590       600       610       620       630
                 |         |         |         |         |         |         |
PctA    TRRIGEIDGMNQVLATATEEQTAVVDSLNMDITEINTLNQEGVENLQATLRACGELETQAGRLRQLVDSFKI
PctB    TGRIAEIDGMNQSVATATEEQTAVVDSLNMDITEINTLNQEGVENLQATLRACGELETQAGRLRQLVDSFKI
PctC    TSRIGEIDSMNQCVATATEEQTAVVDSLNMDITEINTLNQEGVENLQATLRACGELETQAGRLRHLVDSFKI
```
