# Supplementary material for: How Bacterial Chemoreceptors Evolve Novel Ligand Specificities
Source: mBio. 2020 Jan 21;11(1):e03066-19. doi: 10.1128/mBio.03066-19 (PMC6974571; doi:10.1128/mBio.03066-19)
Supplement: TABLE S1 [file mBio.03066-19-st001.pdf]

Table S1) Structural alignment of PctA/B/C-LBD structures. A) Alignment of structures amongst each other. RMSD values from alignments using the DALI algorithm of chains A of PctA-LBD and PctB-LBD and the seven chains of the PctC-LBD asymmetric unit. For the latter structure these chains were in particular: chains A and B with GABA bound to an analogous position as the different amino acids in PctA-LBD and PctB-LBD, chains C, D and E containing GABA bound at the entrance of the binding pocket; chain F with acetate in the membrane distal pocket and chain G with an un-occupied binding site.

	**PctA-LBD**			**PctB-LBD**		**PctC-LBD**						
	Ile	Trp	Met	Arg	Gln	A-GABA	B-GABA	C-GABAm	D-GABAm	E-GABAm	F-Act	G
PctA-LBD/L-Ile	0	0.578	0.587	0.878	1.077	1.249	1.278	2.551	2.574	2.519	2.244	1.715
PctA-LBD/L-Trp		0	0.424	0.977	1.147	1.351	1.388	2.504	2.516	2.468	2.175	1.642
PctA-LBD/L-Met			0	1.158	1.243	1.290	1.335	2.605	2.605	2.567	2.225	1.639
PctB-LBD/L-Gln				0	0.635	1.448	1.407	2.545	2.588	2.505	2.370	1.820
PctB-LBD/L-Arg					0	1.540	1.481	2.521	2.592	2.510	2.349	1.788
PctC-LBD/GABA (A)						0	0.290	2.028	1.993	1.965	1.710	1.239
PctC-LBD/GABA (B)							0	2.058	2.044	2.014	1.673	1.543
PctC-LBD/GABA (C)								0	0.367	0.322	0.697	0.614
PctC-LBD/GABA (D)									0	0.341	0.552	0.633
PctC-LBD/GABA (E)										0	0.616	0.605
PctC-LBD/acetate (F)											0	0.732
PctC-LBD (G)												0

B) Alignment with homologous structures deposited in the pdb databank. Listed are the closest matches from a DALI alignment of structures in the pdb database with the structure of PctA-LBD_L-Ile.

	PDB	Z	RMSD	% ID	organism	Prot family	ligands	Ref.
PctB	5lt9	31.6	1.4	70	*P. aeruginosa*	chemoreceptor	5 amino acids	This work
Mlp37	5ave	27.3	2.4	32	*Vibrio cholerae*	chemoreceptor	9 amino acids/taurine	(1)
PctC	5ltv	25.5	2.5	54	*P. aeruginosa*	chemoreceptor	GABA, His, Pro	This work
Mlp24	6ior	23.4	2.9	31	*Vibrio cholerae*	chemoreceptor	11 amino acids	(2)
McpX	6d8v	21.4	2.2	19	*Sinorhizobium meliloti*	chemoreceptor	Quaternary amines	(3)
Z3	3lib	20.9	2.3	22	*Methanosarcina mazei*	histidine kinase	?	(4)
Tlp1	4wy9	20.5	2.8	17	*Campylobacter jejuni*	chemoreceptor	L-Asp[1]	(5)
Z6	3lic	19.9	2.9	19	*Shewanella oneidensis*	histidine kinase	-	(4)
Z2	3li8	19.8	2.9	22	*M. mazei*	histidine kinase	-	(4)
Z16	3lif	19.4	2.6	14	*Rhodopseudomonas palustris*	histidine kinase	-	(4)
TlpQ	6fu4	19.4	2.7	18	*P. aeruginosa*	chemoreceptor	Histamine and 5 polyamines	(6)
TlpA	6e0a	19.0	3.2	17	*Helicobacter pylori*	chemoreceptor	L-Arg	(7)
vcDctB	3by9	18.5	3.0	15	*V. cholerae*	histidine kinase	Various C_4-dicarboxylates	(8)
smDctB	2zbb	18.3	2.7	17	*Sinorhizobium meliloti*	histidine kinase	C3- and C_4-dicarboxylates	unpublished
TlpC	5wbf	18.1	2.8	15	*H. pylori*	chemoreceptor	lactate	(9)
McpU	6f9g	18.0	2.4	18	*P. putida*	chemoreceptor	Histamine and polyamines	(10)
Tlp3	4xm q	17.3	3.4	19	*C. jejuni*	chemoreceptor	Ile, Lys, Arg, purine, malic acid, fumarate, succinate glucosamine, thiamine	(11)
vpHK1S-Z8	3lid	16.0	3.6	13	*Vibrio parahaemolyticus*	histidine kinase	-	(4)
HK4	3t4k	14.5	3.5	16	*Arabidopsis thaliana*	histidine kinase	cytokines	(12)

[1] This receptor mediates chemotaxis to L-Asp, but an indirect binding mechanism was proposed (5).

C) Relative positions of the four amino acids conserved in the ligand binding pocket of the three paralogs. Shown are all atom rmsd values (Å) for each of the amino acids in pairwise structural alignments. Structures in complex with L-Ile (PctA) and L-Gln (PctB) were used in these alignments.

amino acid[1]	PctA-PctB	PctA-PctC	PctB-PctC
Y121(124)	0.23	0.80	0.89
R126(129)	0.43	1.15	1.05
W128(131)	0.47	1.04	1.03
D173(176)	0.21	0.86	0.92

[1] the residue number in PctC is shown in brackets.

References

1. Nishiyama S, Takahashi Y, Yamamoto K, Suzuki D, Itoh Y, Sumita K, Uchida Y, Homma M, Imada K, Kawagishi I. 2016. Identification of a *Vibrio cholerae* chemoreceptor that senses taurine and amino acids as attractants. Sci Rep 6:20866.
2. Takahashi Y, Nishiyama SI, Sumita K, Kawagishi I, Imada K. 2019. Calcium Ions Modulate Amino Acid Sensing of the Chemoreceptor Mlp24 of *Vibrio cholerae*. J Bacteriol 201.
3. Shrestha M, Compton KK, Mancl JM, Webb BA, Brown AM, Scharf BE, Schubot FD. 2018. Structure of the sensory domain of McpX from *Sinorhizobium meliloti*, the first known bacterial chemotactic sensor for quaternary ammonium compounds. Biochem J 475:3949-3962.
4. Zhang Z, Hendrickson WA. 2010. Structural characterization of the predominant family of histidine kinase sensor domains. J Mol Biol 400:335-53.
5. Machuca MA, Liu YC, Beckham SA, Gunzburg MJ, Roujeinikova A. 2016. The crystal structure of the tandem-PAS sensing domain of *Campylobacter jejuni* chemoreceptor Tlp1 suggests indirect mechanism of ligand recognition. J Struct Biol 194:205-13.
6. Corral-Lugo A, Matilla MA, Martin-Mora D, Silva Jimenez H, Mesa Torres N, Kato J, Hida A, Oku S, Conejero-Muriel M, Gavira JA, Krell T. 2018. High-Affinity Chemotaxis to Histamine Mediated by the TlpQ Chemoreceptor of the Human Pathogen *Pseudomonas aeruginosa*. MBio 9.
7. Sweeney EG, Perkins A, Kallio K, James Remington S, Guillemin K. 2018. Structures of the ligand-binding domain of *Helicobacter pylori* chemoreceptor TlpA. Protein Sci 27:1961-1968.
8. Cheung J, Hendrickson WA. 2008. Crystal structures of C4-dicarboxylate ligand complexes with sensor domains of histidine kinases DcuS and DctB. J Biol Chem 283:30256-65.
9. Machuca MA, Johnson KS, Liu YC, Steer DL, Ottemann KM, Roujeinikova A. 2017. *Helicobacter pylori* chemoreceptor TlpC mediates chemotaxis to lactate. Sci Rep 7:14089.
10. Gavira JA, Ortega A, Martin-Mora D, Conejero-Muriel MT, Corral-Lugo A, Morel B, Matilla MA, Krell T. 2018. Structural Basis for Polyamine Binding at the

dCACHE Domain of the McpU Chemoreceptor from *Pseudomonas putida*. J Mol Biol 430:1950-1963.

11. Liu YC, Machuca MA, Beckham SA, Gunzburg MJ, Roujeinikova A. 2015. Structural basis for amino-acid recognition and transmembrane signalling by tandem Per-Arnt-Sim (tandem PAS) chemoreceptor sensory domains. Acta Crystallogr D Biol Crystallogr 71:2127-36.
12. Hothorn M, Dabi T, Chory J. 2011. Structural basis for cytokinin recognition by *Arabidopsis thaliana* histidine kinase 4. Nat Chem Biol 7:766-8.
