# Supplementary material for: How Bacterial Chemoreceptors Evolve Novel Ligand Specificities
Source: mBio. 2020 Jan 21;11(1):e03066-19. doi: 10.1128/mBio.03066-19 (PMC6974571; doi:10.1128/mBio.03066-19)

Text S1) Study of the size of the ligand binding site and ligand orientation in PctA/PctB/PctC-LBDs structures based on MD simulations.

I) Study of size of ligand binding site

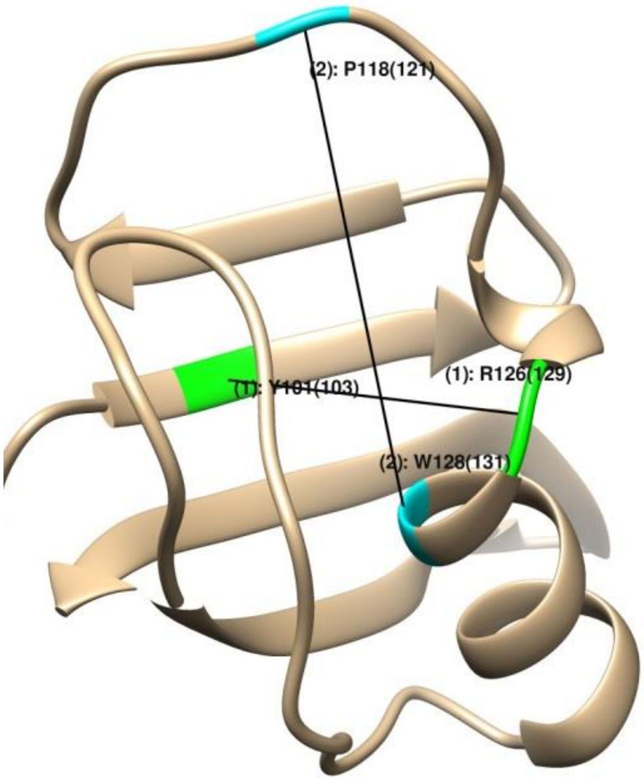

Illustration of distances measured. Amino acid numbering of PctA and PctB is provided and the corresponding residue in PctC is given in parentheses.

	**Width of binding pocket**		**Height of binding pocket**	
	Y101(103)-R126(129)		P118(121)-W128(131)	
	Mean distance (Å)	SD (Å)	Mean distance (Å)	SD (Å)
PctA-LBD	13.08	0.59	17.65	1.64
PctA-LBD_Ile	12.90	0.48	17.08	0.79
PctA-LBD_Met	13.18	0.34	16.94	0.77
PctA-LBD_Trp	13.19	0.40	17.35	0.79
PctB-LBD	13.47	0.55	18.17	0.84
PctB-LBD_Arg	12.97	0.40	16.74	0.69
PctB-LBD_Gln	12.83	0.34	17.86	0.87
PctC-LBD	13.65	1.12	17.65	1.31
PctC-LBD_GABA	12.69	0.89	17.75	1.24

Data were calculated during 10 ns MD simulations and the average distance and standard deviation (SD) between Cα of the residues are indicated in this Table.

II) Study of ligand orientation

We estimated the typical orientation of ligands in the ligand-binding pocket of the paralogs by calculating three types of angles.

A) The angle between two vectors made of CA atoms of Asp173 and Gly168 in PctA/PctB (Asp176 and Gly176 in PctC) and ligand atoms N and C (CD in case of GABA). This parameter determines the alignment of the backbone atoms of bound molecules **along β-strand β6**.

B) The angle between two vectors made of CA atoms of Trp128 and Ala132 in PctA/PctB (Trp131 and Ala135 in PctC) and ligand atoms N and C (CD in case of GABA). This parameter determines the alignment of the ligands **along the α-helix α4**.

C) The angle between two planes made of CA atoms of Asp173, Gly168 and Trp128 in PctA/PctB (Asp176, Gly176, and Trp13 in PctC) and ligand atoms N, C, and CA (N, CD, and the geometric average between CA and CG in case of GABA). This parameter determines the **rotation** of ligands in the ligand-binding pocket.

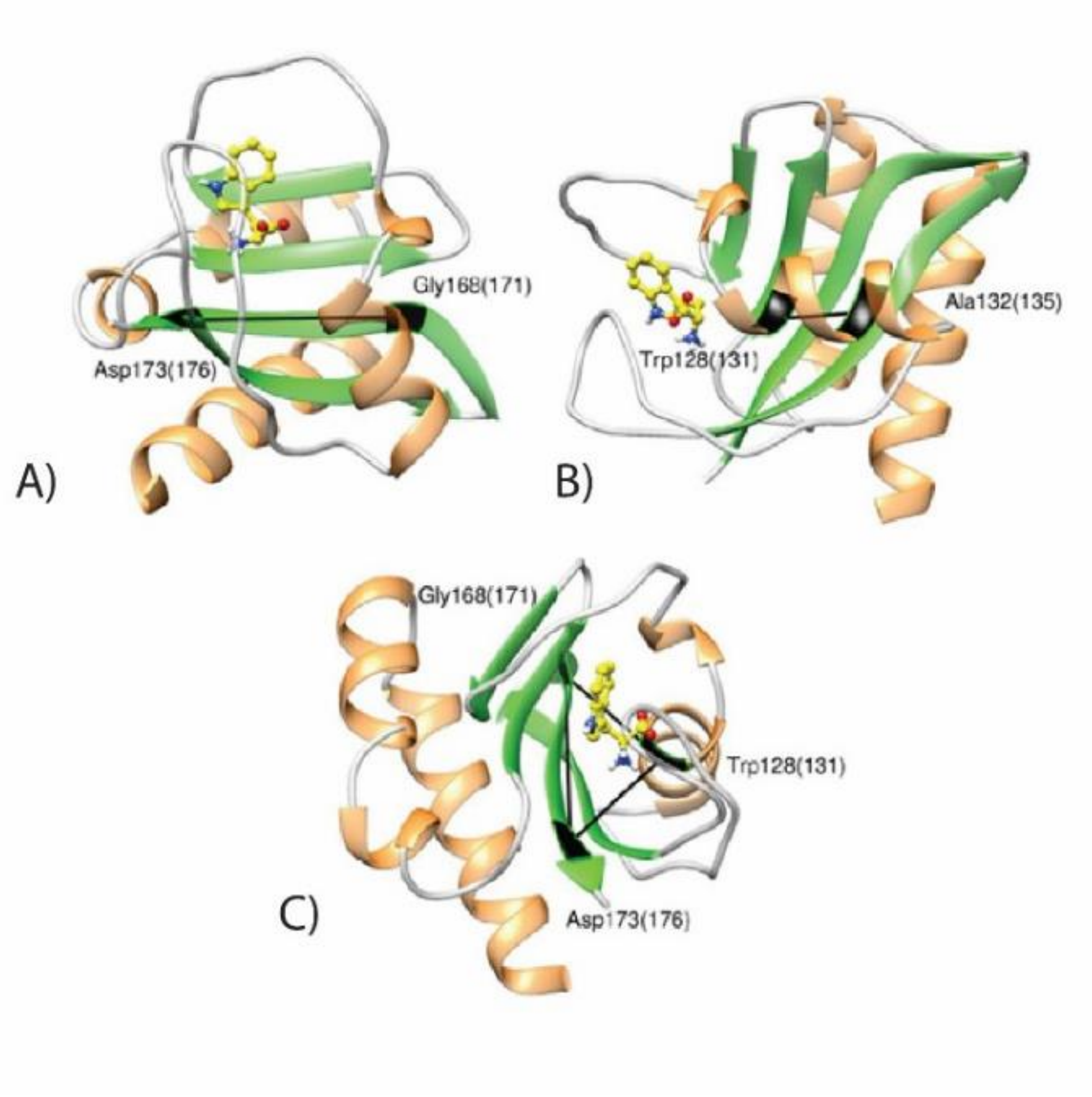

The data on the angles were collected from 539 snapshots extracted from MD simulation. Using the Shapiro-Wilk test and exploring quantile-quantile plots we established that the distribution of the angles deviates from normal. Therefore, to see if there are statistically significant differences in angles between PctA, PctB and PctC we ran the non-parametric Kruskal-Wallis test and post hoc Wilcoxon rank-sum tests with Benjamini-Hochberg correction for multiple testing. The tests were performed using both mean angle values and angle values of the ligands that are closest in size among those that were used in simulation for each paralog. Results showed statistically significant differences in the ligand orientation inside the pocket between the three paralogs (see below).

A) Angle along β-strand β6

Results of the tests for angle values of the amino acids closest in size.

Kruskal-Wallis chi-squared = 372.89, df = 2, p-value < 2.2e-16.

p-values of Wilcoxon rank-sum tests with Benjamini-Hochberg correction:

	PctA_Met	PctB_Gln
PctB_Gln	< 2e-16	-
PctC_GABA	< 1.7e-12	1.4e-07

Results of the tests for mean angle values.

Kruskal-Wallis chi-squared = 382.54, df = 2, p-value < 2.2e-16.

p-values of Wilcoxon rank-sum tests with Benjamini-Hochberg correction:

	PctA	PctB
PctB	< 2e-16	
PctC	8.4e-08	1.2e-05

B) Angle along α-helix α4

Results of the tests for angle values of the amino acids closest in size.

Kruskal-Wallis chi-squared = 379.09, df = 2, p-value < 2.2e-16.

p-values of Wilcoxon rank-sum tests with Benjamini-Hochberg correction:

	PctA_Met	PctB_Gln
PctB_Gln	< 2e-16	-
PctC_GABA	< 2e-16	7.6e-08

Results of the tests for mean angle values.

Kruskal-Wallis chi-squared = 371.38, df = 2, p-value < 2.2e-16.

p-values of Wilcoxon rank-sum tests with Benjamini-Hochberg correction:

	PctA	PctB
PctB	< 2e-16	-
PctC	3.3e-09	1.0e-14

C) Rotation

Results of the tests for angle values of the amino acids closest in size.

Kruskal-Wallis chi-squared = 900.39, df = 2, p-value < 2.2e-16.

p-values of Wilcoxon rank-sum tests with Benjamini-Hochberg correction:

	PctA_Met	PctB_Gln
PctB_Gln	< 2e-16	-
PctC_GABA	< 2e-16	< 2e-16

Results of the tests for mean angle values.

Kruskal-Wallis chi-squared = 974.82, df = 2, p-value < 2.2e-16.

p-values of Wilcoxon rank-sum tests with Benjamini-Hochberg correction:

	PctA	PctB
PctB	< 2e-16	-
PctC	< 2e-16	< 2e-16

Graphical illustration of Results

A

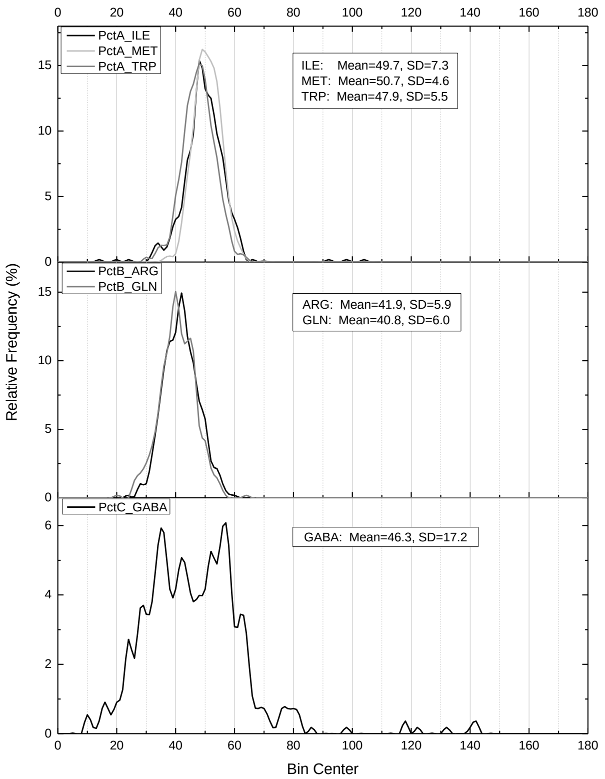

B

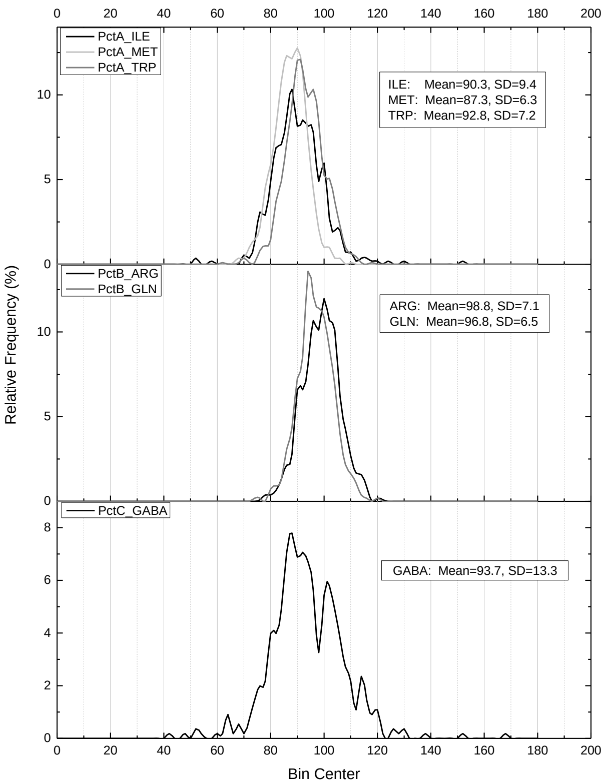

C

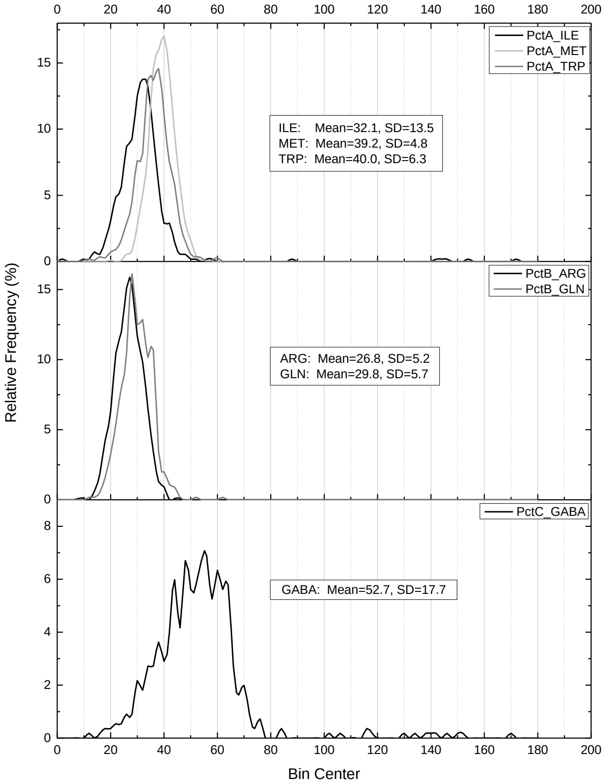

Supplement: TEXT S1 [file mBio.03066-19-s0001.pdf]
