# Supplementary material for: How Bacterial Chemoreceptors Evolve Novel Ligand Specificities
Source: mBio. 2020 Jan 21;11(1):e03066-19. doi: 10.1128/mBio.03066-19 (PMC6974571; doi:10.1128/mBio.03066-19)
Supplement: TABLE S2 [file mBio.03066-19-st002.pdf]

Table S2) Glide XP docking scores obtained for PctA-LBD, PctB-LBD and PctC-LBD for the best ligand candidates. Ligands indicated with an asterisk * were tested experimentally by Isothermal Titration Calorimetry.

PctA-LBD		PctB-LBD		PctC-LBD	
**Membrane Distal Bundle**					
L-Trp	-13.74	L-Arg	-10.38	GABA	-12.32
L-Ile	-12.74	L-Gln	-9.37		
L-Met	-11.53				
**Membrane Proximal Bundle**					
3-Methylbutyrolactone	-6.53	N1-Methyl-4-pyridone-3-carboxamide	-4.47	2-aminoethoxy-hydroxy-phosphoryl	-6.05
5-Hydroxymethyl-2(5H)-furanone	-6.51	Ethanolamine	-4.43	Nadide	-6.04
γ-Butyrolactone *	-6.51	Propylene oxide	-4.31	1,6-Digalloyl-beta-D-glucopyranose	-5.92
γ-Caprolactone *	-6.41	1-Amino-2-propanol	-4.29	6-Amino-1H-purin-2(7H)-one sulfate	-5.91
Chloral hydrate *	-6.28	2-Hydroxyacetamide	-4.21	Neopterin	-5.85
Dihydrofuran-3(2H)-one	-6.16	Acetamide	-4.11	Histamine	-5.81
