# Supplementary material for: How Bacterial Chemoreceptors Evolve Novel Ligand Specificities
Source: mBio. 2020 Jan 21;11(1):e03066-19. doi: 10.1128/mBio.03066-19 (PMC6974571; doi:10.1128/mBio.03066-19)
Supplement: TABLE S3 [file mBio.03066-19-st003.pdf]

Table S3) Conditions used for protein crystallization.

Protein	**PctA-LBD**			**PctB-LBD**		**PctC-LBD**
Ligand	L-Ile	L-Trp	L-Met	L-Gln	L-Arg	GABA
Precipitant	2.8 M NH_4/sulfate, 0.1 M Na/acetate	2.0 M Na/formate, 0.1 M Na/acetate	2.0 M Na/formate, 0.1 M Na/acetate	1.7 M NH_4/sulfate, 3.5% (w/v) PEG 400, 0.1 M Na/Hepes	1.25 M Na/citrate, 0.1 M Na/Hepes	2.0 M NH_4/sulfate, 0.1 M Tris/HCl
pH	pH 5.0	pH 4.6	pH 4.6	pH 7.5	pH 7.5	pH 8.5
